# Glycoengineering HIV-1 Env creates ‘supercharged’ and ‘hybrid’ glycans to increase neutralizing antibody potency, breadth and saturation

**DOI:** 10.1371/journal.ppat.1007024

**Published:** 2018-05-02

**Authors:** Ema T. Crooks, Samantha L. Grimley, Michelle Cully, Keiko Osawa, Gillian Dekkers, Kevin Saunders, Sebastian Rämisch, Sergey Menis, William R. Schief, Nicole Doria-Rose, Barton Haynes, Ben Murrell, Evan Mitchel Cale, Amarendra Pegu, John R. Mascola, Gestur Vidarsson, James M. Binley

**Affiliations:** 1 San Diego Biomedical Research Institute, San Diego, California, United States of America; 2 Vaccine Research Center, National Institute of Allergy and Infectious Diseases, National Institutes of Health, Bethesda, Maryland, United States of America; 3 Department of Experimental Immunohematology, Sanquin Research and Landsteiner Laboratory, Academic Medical Center, University of Amsterdam, Amsterdam, The Netherlands; 4 Duke University Medical Center, Durham, North Carolina, United States of America; 5 The Scripps Research Institute, Department of Immunology and Microbial Science, La Jolla, California, United States of America; 6 IAVI Neutralizing Antibody Center, The Scripps Research Institute, Department of Immunology and Microbial Science, La Jolla, California, United States of America; 7 Ragon Institute, Cambridge, Massachusetts, United States of America; 8 Department of Medicine, University of California San Diego, San Diego, California, United States of America; King’s College London, UNITED KINGDOM

## Abstract

The extensive glycosylation of HIV-1 envelope (Env) glycoprotein leaves few glycan-free holes large enough to admit broadly neutralizing antibodies (bnAb). Consequently, most bnAbs must inevitably make *some* glycan contacts and avoid clashes with others. To investigate how Env glycan maturation regulates HIV sensitivity to bnAbs, we modified HIV-1 pseudovirus (PV) using various glycoengineering (GE) tools. Promoting the maturation of α-2,6 sialic acid (SA) glycan termini increased PV sensitivity to two bnAbs that target the V2 apex and one to the interface between Env surface gp120 and transmembrane gp41 subunits, typically by up to 30-fold. These effects were reversible by incubating PV with neuraminidase. The same bnAbs were unusually potent against PBMC-produced HIV-1, suggesting similar α-2,6 hypersialylated glycan termini may occur naturally. Overexpressing β-galactosyltransferase during PV production replaced complex glycans with hybrid glycans, effectively 'thinning' trimer glycan coverage. This increased PV sensitivity to some bnAbs but ablated sensitivity to one bnAb that depends on complex glycans. Other bnAbs preferred small glycans or galactose termini. For some bnAbs, the effects of GE were strain-specific, suggesting that GE had context-dependent effects on glycan clashes. GE was also able to increase the percent maximum neutralization (i.e. saturation) by some bnAbs. Indeed, some bnAb-resistant strains became highly sensitive with GE—thus uncovering previously unknown bnAb breadth. As might be expected, the activities of bnAbs that recognize glycan-deficient or invariant oligomannose epitopes were largely unaffected by GE. Non-neutralizing antibodies were also unaffected by GE, suggesting that trimers remain compact. Unlike mature bnAbs, germline-reverted bnAbs avoided or were indifferent to glycans, suggesting that glycan contacts are acquired as bnAbs mature. Together, our results suggest that glycovariation can greatly impact neutralization and that knowledge of the optimal Env glycoforms recognized by bnAbs may assist rational vaccine design.

## Author summary

Here we engineered various changes in the sizes and shapes of sugars that decorate HIV surface spike proteins and tested the effects of these changes on virus susceptibility to neutralizing antibodies. In so doing, we were able to define the optimal Env-sugars recognized by prototype bnAbs that recognize various canonical epitope clusters on Env spike proteins. Some bnAbs preferred spike proteins decorated with large, complex glycans. Others preferred smaller glycans that improved their access to underlying protein targets. For similar reasons, germline-reverted versions of bnAbs were also generally more effective when the glycans were small. In some cases, bnAbs acquired an ability to bind to sugars as they matured. A comparison of viruses generated in cell lines and primary cells revealed large differences in bnAb sensitivity, raising questions about clinical relevance of cell line-produced virus for checking vaccine responses and, moreover, the use of these cell lines for manufacturing vaccines. Overall, just as car engines may be modified to be supercharged or hybrid for increased power or efficiency, the sugars of HIV coat proteins may also need to be engineered as 'supercharged' and 'hybrid' or otherwise modified in rational vaccine designs to optimize bnAb recognition.

## Introduction

Neutralizing antibodies (nAbs) are likely to be an essential part of the immunity conferred by an effective HIV-1 vaccine [1]. NAbs interfere with HIV-1 infection by binding to functional Envelope glycoprotein (Env) spikes consisting of gp120/gp41 trimers arrayed on virion surfaces, thereby blocking receptor engagement and/or membrane fusion. Env trimer surfaces are populated by a dense glycan network that constitutes ~50% of its mass [2–5]. Since anti-glycan Abs are regulated by immunological tolerance, glycans provide a formidable defense against nAbs that, at least initially, must attempt to navigate past to reach underlying protein epitopes [6–8]. Although the space between glycans is sufficient for access by single immunoglobulin domain ligands (e.g., soluble CD4 and llama Abs) and by bovine Abs with very long, protruding heavy chain third complementarity determining loops (CDRH3s) [9–11], glycan-free spaces are typically insufficient for human Abs, that consist of two immunoglobulin chains and less protrusive CDRH3s. Structural and glycan array studies reveal that some bnAbs overcome this problem by contacting composite protein-glycan epitopes [4].

**Fig 1:**
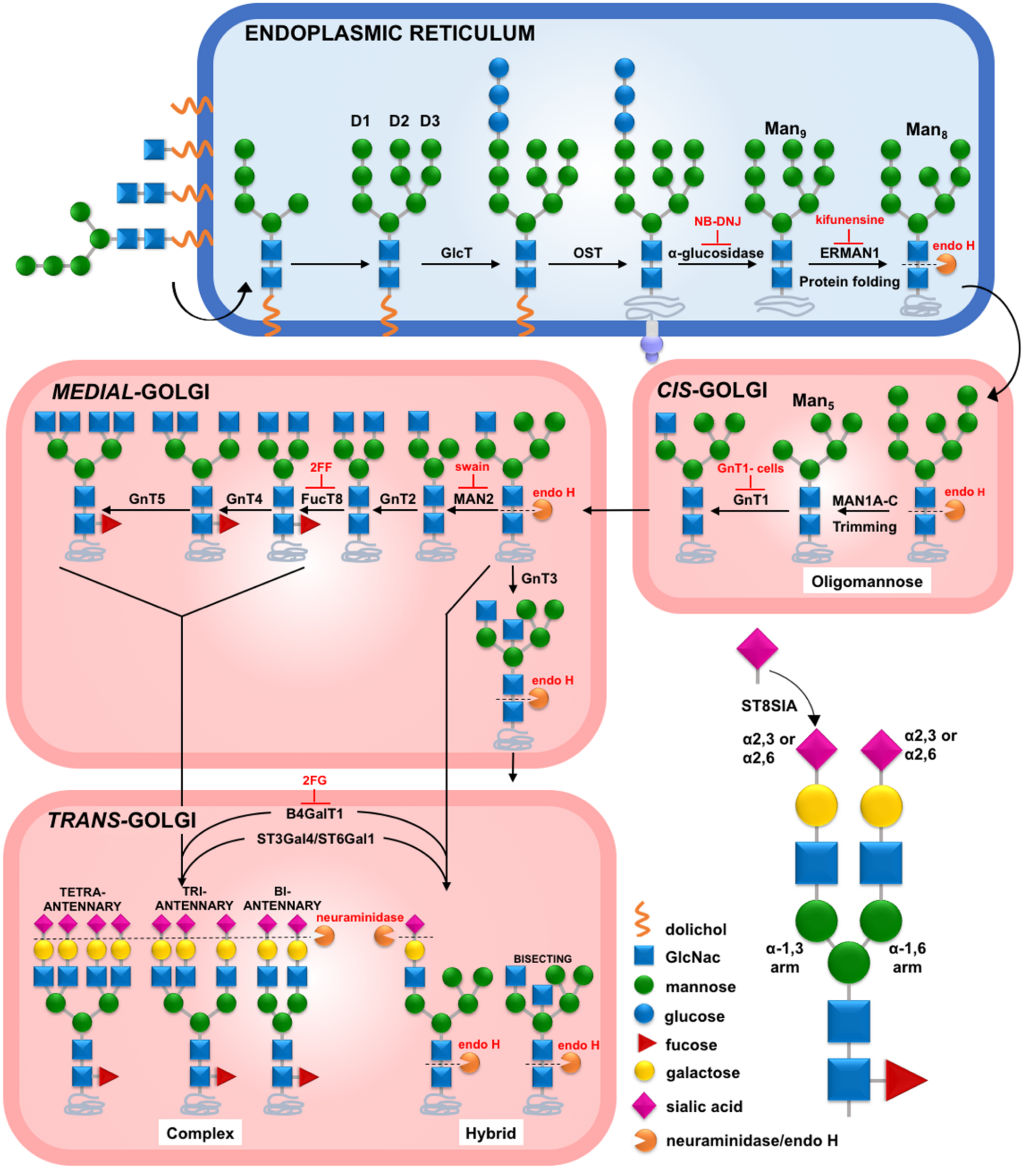
N-linked glycosylation and GE. A dolichol phosphate-linked precursor consisting of 2 membrane-linked N-acteylglucosamine (GlcNac) moieties and 5 mannose (Man) forms on the cytoplasmic surface of the endoplasmic reticulum (ER), then flips to the lumen. Further mannose moieties are added to create 3 termini (D1-D3), to which glucose moieties are added by glucosyltransferase (GlcT). This is transferred to Asn-X-Ser/Thr sequons of a nascent protein by oligosaccharyltransferase (OST). The 3 terminal glucose residues are then removed by α-glucosidase to form Man_9_GlcNAc_2_ (Man9)—a step that is inhibited by N-Butyldeoxynojirimycin (NB-DNJ). The terminal D2 mannose is then cleaved by α-mannosidase 1 (ERMAN1)—a step that can be inhibited by kifunensine. In the cis-Golgi, mannose is trimmed by α-mannosidases 1A, 1B and 1C (MAN1A-C) to form Man_5_GlcNAc_2_. GlcNAc is then transferred to the α-1,3 D1 arm by N-acetylglucosaminyltransferase 1 (GNT1; inactive in GNT1- cells). In the medial Golgi, there is a bifurcation in the pathway. In one fork, D2 and D3 mannose subunits are removed by α-mannosidase II (MAN2)—a step that is blocked by swainsonine (swain). GlcNAc moieties may then be added to the trimmed α-1,6 arm by GNT2 to initiate a biantennary glycan, followed by the addition of a core fucose moiety by fucosyltransferase (FUCT8), a step that is blocked by 2-deoxy-2-fluoro-1-fucose (2FF). Further GlcNAc termini may then be added by GNT4 and GNT5 to form tri- and tetra-antennary glycans. These may be galactosylated by β-1,4 galactosyltransferases (B4GALT1), a step that is blocked by 2-deoxy-2-fluoro-d-galactose (2FG). Terminal SA may then be added by β-galactoside α-2,3-sialyltransferase (ST3GAL4) or β-galactoside α-2,6-sialyltransferase (ST6GAL1). Polysialic chains may form by the addition of α-2,8-linked SA by α-2,8-sialyltransferase (ST8SIA4). SAs can be cleaved by NA. In the alternative fork, α-1,6 arm mannose is not removed, but the α-1,3 arm may be modified with galactose and SA, forming hybrid glycans, sometimes with the addition of a bisecting GlcNAc subunit by GNT3 and no fucosylation. Non-fucosylated high mannose and hybrid glycans can be cleaved by endoglycosidase H (endo H).

HIV-1 Env's unparalleled sequence diversity presents a daunting challenge for vaccinologists aiming to induce broadly neutralizing antibodies (bnAbs). The heterogeneity of its surface glycans could add an additional layer of difficulty. In mammals, N-linked glycosylation involves >700 genes that can impart a plethora of carbohydrate structures to Asn-X-Ser/Thr sequons (where X can be any amino acid except for proline) [12]. Glycosylation (summarized in Fig 1) begins in the endoplasmic reticulum, where an oligomannose glycan precursor (Glc_3_Man_9_GlcNAc_2_; Glc = glucose, Man = mannose, GlcNAc_2_ = N-acetylglucosamine) is transferred to nascent proteins prior to folding (Fig 1: top row, fourth glycan from left). Trimming of mannose termini results in Man_5_GlcNAc_2_, the simplest oligomannose glycan, which may then be modified to form a variety of hybrid or complex glycans that may be fucosylated, galactosylated, sialylated and/or bisected by a central GlcNAc moiety (the latter is modeled in Fig 1: bottom row, rightmost hybrid glycan).

The 75–105 N-linked glycoforms on the surface of HIV-1 Env trimers range from untrimmed high mannose glycans to complex, multiantennary glycans. One key regulator of their maturation state is the cellular expression of enzymes that catalyze each step (Fig 1), which depends on factors including host genetics, age, infection, and pregnancy [13, 14]. Although early mannose trimming is usually efficient, later steps such as galactosylation and sialylation may be inefficient, so that some complex and hybrid glycans may incompletely mature and lack these termini. Further variation can arise from the covalent bond angle of terminal sialic acid (SA) moieties, which may be predominantly α-2,3 or α-2,6-linked in different species, tissues and cell lines [15–18]. For example, peripheral blood mononuclear cell (PBMC)-derived HIV-1 Env is modified mostly with α-2,6-linked SAs, whereas that produced in human embryonic kidney (HEK) 293T or Chinese hamster ovary (CHO) cells bears mostly or exclusively α-2,3-linked SAs, respectively [17, 19, 20]. Furthermore, 293T and Jurkat cell lines impart a higher proportion of high mannose and hybrid glycans than CHO cells [15]. In humans, α-2,8-linked SA may be linked to α-2,3 or α-2,6-linked SA, usually in neuronal tissue (Fig 1).

The density of surface glycans is so great in some Env domains that α-mannosidase, like bnAbs, has difficulty in gaining access due to steric constraints. This results in an unusually high proportion of immature oligomannose glycoforms (Man_5_GlcNAc_2_ –Man_9_GlcNAc_2_), including an oligomannose patch common to all forms of Env [17, 21, 22]. In general, as Env sequons "compete" for glycan addition and modification, each may variably become occupied by oligomannose, hybrid or complex glycans or, due to steric competition with neighboring sequons, may occasionally remain unoccupied ("sequon skipping"). Together, the above factors contribute to considerable Env glycodiversity [21, 23].

HIV-1 bnAbs fall into 5 distinct epitope clusters: V2 apex, V3-glycan, CD4 binding site (CD4bs), gp120-gp41 interface and membrane-proximal external region (MPER), whose epitopes collectively cover a large portion of the trimer's exposed surface [2, 4, 24–29]. Since most of these bnAbs make some glycan contacts, it appears possible that glycodiversity could modulate their activities [30].

V2 apex-specific bnAbs include at least five families (PG9, CAP256, CH01, PGT145 and PCT64-35S) that exhibit unusually long (>24 amino acid) anionic CDRH3s that project outward to penetrate Env’s glycan shield and reach underlying protein [31–41]. In contrast, another V2 apex lineage represented by VRC38.01 uses a 16AA non-protruding CDRH3 that binds via side-chain to side-chain contacts [42]. These bnAbs contact the positively charged strand C and conserved glycans, typically at positions N156 and N160 [18, 21, 32, 34, 39, 42]. Previous studies showed that PG9 engages SA termini [39, 43], recognizing α-2,3 SAs in one array [36], but preferentially binding α-2,6 SAs in the context of an intact V1V2 domain [4, 39]. CAP256.09 also binds α-2,6 SAs [4, 40]. In contrast, CH01 recognizes mannosylated V2 peptides [44]. PGT145 does not bind in glycan arrays and appears to be largely insensitive to glycan changes [4]. V2 bnAbs may also ‘clash’ with some glycans. For example, glycans at position 130 of the V1 and C-terminal V2 (V2’) of some strains can limit V2 bnAb sensitivity [42, 45]. Furthermore, productive bnAb-glycan contacts or clashes may be influenced by the glycodiversity mentioned above, resulting in non-sigmoidal neutralization curves and sub-saturating neutralization, even with excess nAb [4, 42, 46, 47].

V3-glycan bnAbs target the intrinsic mannose patch, usually centered around the N332 glycan [37, 48–52]. In the absence of this glycan, proximal glycans, e.g. N137, N156, N295 and N301 can contribute to binding [53]. In some scenarios, however, glycan clashes may regulate neutralization [30]. Some of these bnAbs also recognize complex glycans [4, 37, 48, 50, 54].

Most CD4bs bnAbs are subject to possible clashes with a "glycan fence" that includes the N276 glycan of loop D and V5 loop glycans that surround the underlying receptor binding site [8, 27, 29, 55, 56]. Changes in the composition or maturation state of the glycan fence may regulate virus sensitivity to CD4bs bnAbs [57]. However, some bnAbs (HJ16 and 179NC75) incorporate the N276 glycan in their epitopes [58, 59] and VRC13 contacts several partially mature glycans in arrays [4].

Gp120-gp41 interface bnAbs exhibit diverse glycan dependencies. 35O22 [60] targets a quaternary epitope involving glycans N88, N230, N241 and N625, and binds oligomannose and complex glycans in arrays [4]. PGT151 recognizes tetra-antennary glycans at positions 611 and 637, with the N448 glycan playing a regulatory role [3, 4, 21, 61]. 3BC176 does not bind to glycan arrays but is sterically impacted by the N88 glycan [62, 63]. Conversely, ACS202 and VRC34.01 depend on the N88 glycan, but the latter is sterically impeded by the N611 glycan [64, 65]. 8ANC195 depends on the N234, N276 and N637 glycans and clashes with the N230 glycan [66–68]. Finally, CAP248-2B binds proximal to interface glycans, but appears to be unaffected by glycoform changes [69].

MPER bnAbs 10E8, 2F5, Z13 and 4E10 are not known to recognize glycans [70–72]. However, partial deglycosylation by PNGase F increases 2F5 and 4E10 affinity, perhaps due to the removal of complex glycans at the trimer base [73]. Incomplete neutralization by bnAb 10Ee8 may be due to glycan heterogeneity, particularly at position N625 [74].

Glycoengineering (GE) methods, including several outlined in Fig 1 can alter glycan maturation state and involve the use of: i) glycosylation inhibitors, ii) glycosyltransferase knockout cell lines, iii) *in vitro* enzyme reactions and iv) glycosyltransferase plasmid co-transfections. To date, only a handful of GE methods have been used to modify HIV-1 [4, 37, 75, 76]. For example, kifunensine, which prevents Man_9_GlcNAc_2_ trimming (Fig 1), decreases HIV-1 sensitivity to some V2 apex mAbs [37, 39, 43, 76, 77], but increases sensitivity to PGT125 and 35O22 [60]. Virus production in a knockout cell line lacking functional N-acetylglucosaminyltransferase I (GNT1-) increases virus sensitivity to some mAbs [75, 76], but decreases PGT151 sensitivity [3, 4, 61, 78]. Aside from these and other anecdotal reports, the effects of GE on HIV-1 neutralization have not been comprehensively investigated. To fill in this knowledge gap, we investigated the effects of 16 GE methods on the sensitivities of 293T cell-produced pseudoviruses (PVs) to a large panel of bnAbs. Some bnAbs were dramatically impacted. PG9 and CAP256.09 were up to ~30-fold more potent against PVs produced with co-transfected α-2,6 sialyltransferase. PGT151 and PGT121 were more potent against PVs with terminal SA removed. 35O22 and CH01 were more potent against PV produced in GNT1- cells. The effects of GE on bnAbs VRC38.01, VRC13 and PGT145 were inconsistent between Env strains, suggesting context-specific glycan clashes. Overexpressing β-galactosyltransferase during PV production 'thinned' glycan coverage, by replacing complex glycans with hybrid glycans. This impacted PV sensitivity to some bnAbs. Maximum percent neutralization by excess bnAb was also improved by GE. Remarkably, some otherwise resistant PVs were rendered sensitive by GE. Germline-reverted versions of some bnAbs usually differed from their mature counterparts, showing glycan indifference or avoidance, suggesting that glycan binding is not germline-encoded but rather, it is gained during affinity maturation. Overall, these GE tools provide new ways to improve bnAb-trimer recognition that may be useful for informing the design of vaccine immunogens to try to elicit similar bnAbs.

## Results

Various GE methods (Fig 1) were used to modify HIV-1 PV Env glycans and then examine the effects on nAb sensitivity. In some cases, we co-transfected plasmids encoding glycosyltransferases or added decoy substrates during PV production in 293T cells. In other cases, PVs were produced in GNT1- knockout cell line or were incubated with neuraminidase (NA) to remove SA termini.

An E168K+N189A JR-FL mutant was used to fully knock in PG9 bnAb sensitivity (K168 is a critical contact; N189A knocks out a competitive glycan). We used a gp41 tail-truncated clone (gp160ΔCT) to increase expression with only a marginal impact on its neutralization sensitivity [79]. For convenience, we refer to this clone as 'JR-FL' henceforth.

It was previously reported that HIV-1 desialylation increases infectivity [80]. However, with the exception of swainsonine, our GE methods, several of which eliminate sialylation, did not enhance infection (S1A Fig). In fact, kifunensine and GNT1- PV infectivities were both <20% of control levels. This discrepancy could be due to our use of PV instead of replicating virus in the cited study, that we performed NA digests *after* rather than *during* virus production, and/or that some glycosylation inhibitors may impact Env expression and, by extrapolation, infectivity.

**Fig 2:**
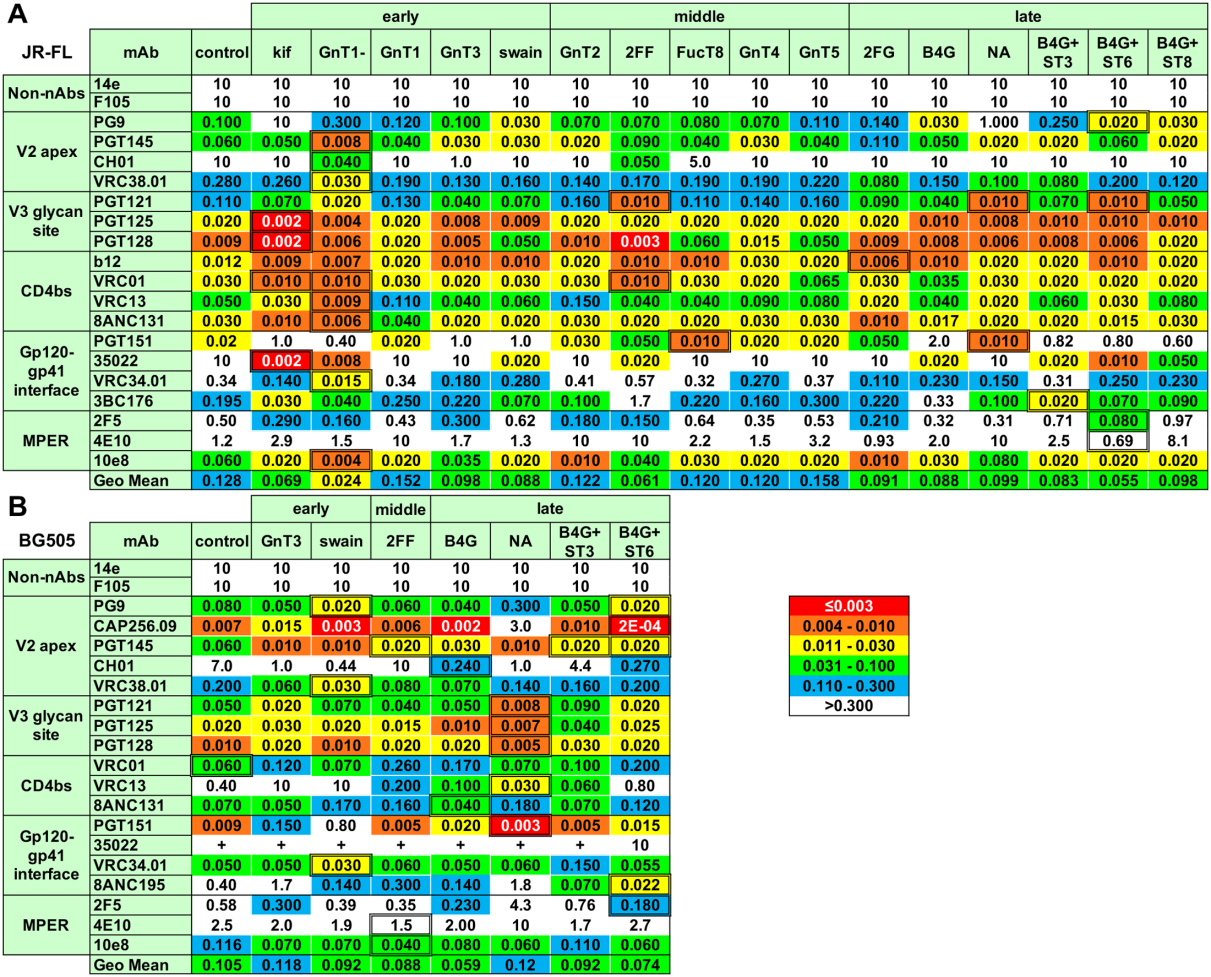
Effects of GE on bnAb neutralization IC50s. Neutralizing IC50s for each control and GE-modified A) JR-FL and B) BG505 PVs are shown in μg/ml. The most sensitive glycoform for each nAb is boxed. JR-FL neutralization assays for mAb 3BC176 used the N88A mutant. Each assay was performed at least in duplicate. In many cases, BG505 neutralization by mAb 35O22 was insufficiently saturating to reach an IC50 and is denoted by a "+". Geometric mean IC50s per treatment are shown, omitting data for 14e, F105 and instances of 35O22 "+". This Fig is linked to Fig 3, S2 and S6 Figs. Abbreviations: B4G = B4GALT1, ST3 = ST3GAL4, ST6 = ST6GAL1, ST8 = ST8SIA4.

**Fig 3:**
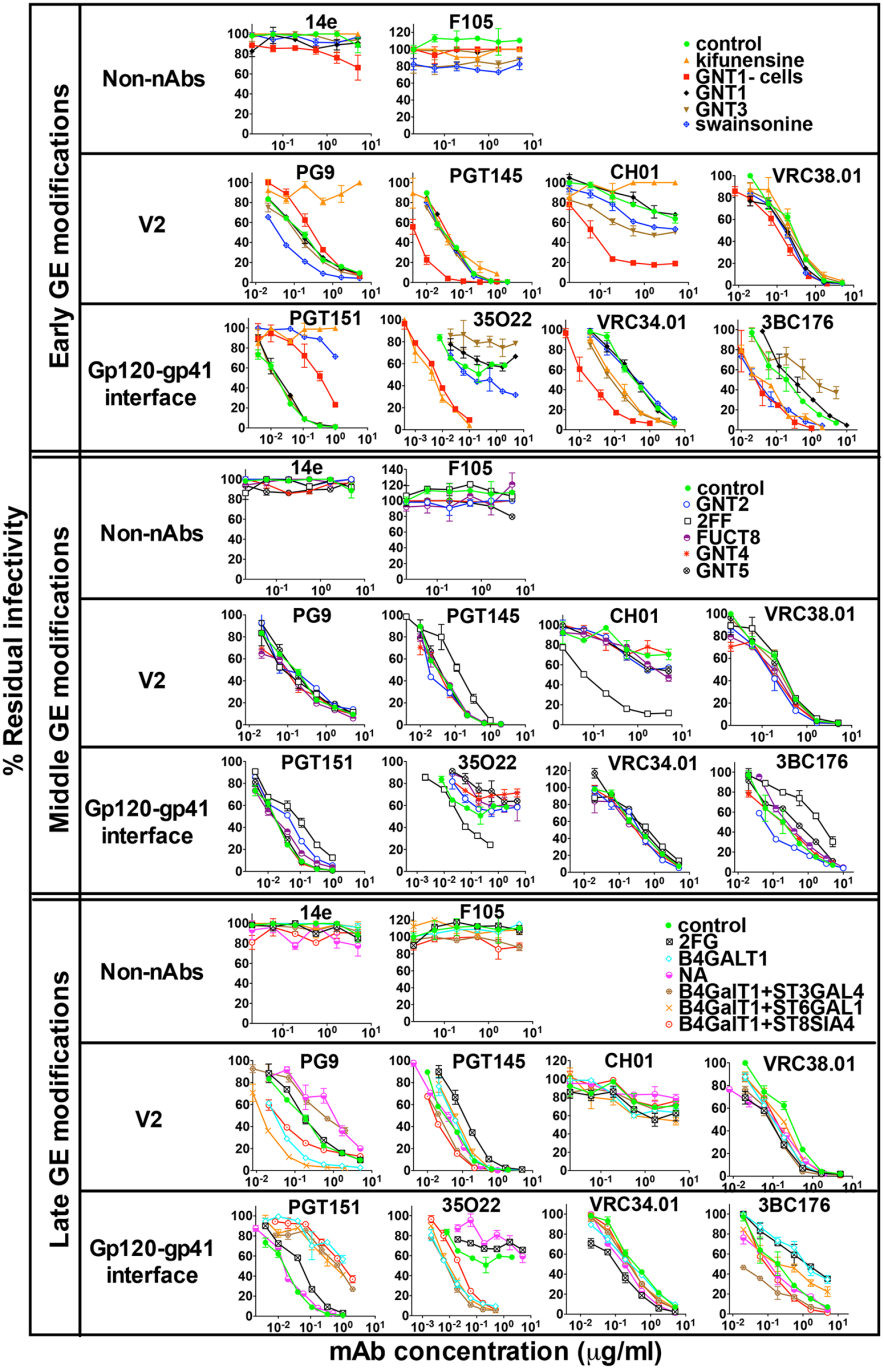
Effects of GE on JR-FL sensitivity to V2 apex and gp120-gp41 interface bnAbs. Non-nAbs 14e and F105 were used to monitor any overt impacts of GE on the tier 2 phenotype. For mAb 3BC176, a JR-FL N88A mutant was used. Results are representative of at least two repeats performed in duplicate. Error bars show standard deviations (SD). IC50s are shown in Fig 2A. GNT3 modification was included in this early set of GE modifications instead of GNT2, as it promotes smaller, hybrid glycans.

The neutralizing activities of a representative panel of bnAbs of the 5 major epitope clusters were next tested against GE-modified PVs. Two non-neutralizing mAbs (non-nAbs) (14e and F105) were also included to monitor for any overt changes in trimer folding. An overview of IC50s as a heat map is shown in Fig 2A. Fig 3 shows the effects of modifying early, middle and late stages of glycosylation on JR-FL sensitivity to V2 apex and gp120-gp41 bnAbs and the non-nAb controls. S2 Fig shows the effects of the same modifications on the 3 other bnAb clusters.

### Early GE tools affect JR-FL sensitivity to V2 apex and gp120/gp41 interface bnAbs

Early GE tools included adding inhibitors kifunensine and swainsonine or co-transfecting plasmids expressing *N*-acetylglucosaminyltransferases 1 and 3 (GNT1 and 3) during PV production in 293T cells. A fifth early GE variant was PV produced in GNT1- cells. The resulting PVs are referred to hereafter as their modification followed by PV. GE-modified PVs were generally resistant to non-nAbs, although the GNT1- PV was mildly sensitive to V3 mAb 14e (Fig 3). Although F105 reduced swainsonine PV infectivity to a plateau at ~80% in the data shown, this was not observed in repeats. Overall, early GE methods did not markedly affected trimer compactness. We next analyzed 4 prototype V2 apex bnAbs: PG9, PGT145, CH01 and VRC38.01. Like PGT145 [4], VRC38.01 was unreactive in oligomannose glycan arrays (S3 Fig). Consistent with previous studies, kifunensine PV was resistant to PG9 and CH01 [37, 42, 77], but had little effect on PGT145 and VRC38.01 [42]. Conversely, GNT1- PV was >100-fold more sensitive to CH01, suggesting that small glycans eliminate binding clashes (Figs 2A and 3). GNT1- PV was also ~10-fold more sensitive PGT145, marginally more sensitive to VRC38.01, and marginally more resistant to PG9. On the other hand, swainsonine, which inhibits D2 and D3 mannose trimming (Fig 1), increased PG9 sensitivity (Figs 2A and 3), suggesting preferential recognition of hybrid glycans [4, 40]. CH01 also preferred swainsonine and GNT3 PVs (Fig 3). Thus, GE here helps to minimize CH01 binding clashes by replacing complex glycans with smaller glycans like Man_5_GlcNAc_2_ (GNT1- PV) or hybrid glycans (swainsonine and GNT3 PVs). The natural scarcity of bisected glycans in previous reports [13, 19, 21] suggests that GNT3 is typically poorly expressed in 293T cells. In contrast, GNT1 plasmid co-transfection had little effect on V2 bnAbs—or indeed on other bnAbs (Figs 2A and 3, S2 Fig), suggesting that natural cellular levels of this enzyme are not limiting.

Gp120-gp41 interface bnAbs were heavily impacted by early GE, and in diverse ways (Fig 3). PGT151 potency was reduced against kifunensine, swainsonine and GNT1- PVs, consistent with the importance of tetra-antennary glycan contacts which are eliminated by these GE methods [4, 61, 78]. In stark contrast, kifunensine and GNT1- PVs were highly sensitive to 35O22, facilitating nearly 100% saturating neutralization and suggesting a preference for high mannose glycans. 35O22 was also modestly more potent against swainsonine PV but was less potent against the GNT3 PV. VRC34.01 was also more potent against GNT1- and kifunensine PVs (Figs 2A and 3), although in this case the GNT1- PV was the most sensitive, suggesting that smaller glycans reduce binding clashes. VRC34.01 sensitivity was also improved against the GNT3 PV, perhaps due to the replacement of complex glycans with smaller hybrid glycans (Fig 1). However, swainsonine, which also promotes hybrid glycans, had no effect, suggesting that fine differences in glycan structures are important. Finally, 3BC176 activity was increased against kifunensine, GNT1- and swainsonine PVs but not GNT3 PV. Thus, 35O22, VRC34.01 and 3BC176 activities were all improved by GE tools that reduce glycan size, albeit with unique patterns that reflect their different binding modes.

Perhaps unsurprisingly, V3-glycan bnAbs were generally only modestly affected by early GE, as they target the intrinsic mannose patch (S2 Fig). The same was true for CD4bs and MPER bnAbs, in this case because they generally target protein epitopes (S2 Fig). Nevertheless, almost all of these bnAbs were more potent against the GNT1- PV, suggesting that smaller glycans help to minimize binding clashes. PGT125 and PGT128 were even more potent against kifunensine PV, consistent with a preference for untrimmed high mannose glycan.

The poor neutralizing activities of CH01 and 35O22 under standard conditions were unexpected (Fig 3). We investigated whether this was assay-related by checking the activities of both mAbs in the CF2 (used throughout this study) and TZM-bl assays. Both mAbs incompletely neutralized the JR-FL PV at high mAb concentrations in both assays (S4 Fig), although the residual infectivity in the CF2 assay plateaued at higher levels. In contrast, VRC01 completely neutralized the PV in both assays, albeit with slightly different IC50s. Although our previous studies have shown these assays yield similar IC50s, modest differences may stem from the higher CCR5 surface density of CF2 cells [79].

### Intermediate GE tools have limited effects on JR-FL sensitivity

Modifying intermediate glycosylation steps did not impact PV resistance to non-nAbs 14e and F105. The effects on bnAbs were generally modest (Figs 2A and 3). However, 2-deoxy-2-fluoro-l-fucose (2FF) (which inhibits core fucosylation; Fig 1) dramatically improved CH01 sensitivity, consistent with the increased GNT1- PV sensitivity (Figs 2A and 3), further underlining a preference for small glycans. The absence of core fucose may facilitate greater glycan flexibility, minimizing clashes. Perhaps for similar reasons, 2FF also markedly increased 35O22 sensitivity and, to a lesser extent, PGT128, b12 and VRC01 sensitivities, but decreased sensitivities to PGT145, PGT151 and 3BC176 (Figs 2A and 3, S2 Fig). As with early GE, mAbs directed to the V3-glycan supersite, CD4bs and MPER epitopes were largely unaffected by other intermediate GE methods (S2 Fig). However, fucosyltransferase 8 (FUCT8) co-transfection modestly decreased PGT128 sensitivity, mirroring the opposite effect of 2FF, suggesting that fucose causes a binding clash. The generally modest impact of FUCT8, GNT4 and GNT5 co-transfections suggests ample cellular levels of these enzymes are already expressed in the host cells [13].

### Late GE tools markedly impact JR-FL sensitivity to V2 apex and gp120/gp41 interface bnAbs

Late GE had no effect on non-nAbs and only mild effects on 3 of the 4 V2 bnAbs (Figs 2A and 3). In contrast, β-1,4-galactosyltransferase 1 (B4GALT1) co-transfection increased PG9 potency by ~10-fold, suggesting that it helps promote the development of differentiated hybrid or complex glycan termini (Fig 1) [4, 39]. Conversely, inhibiting galactosylation with 2-deoxy-2-fluoro-d-galactose (2FG) had little impact on V2 bnAbs, except for a moderate decrease in PGT145 sensitivity (Figs 2A and 3).

Sialylation depends on effective galactosylation (Fig 1). Since the latter may be limiting, we promoted Env sialylation by co-transfecting various sialyltransferase plasmids together with B4GALT [13]. Co-transfection of β-galactoside α-2,6-sialyltransferase 1 (ST6GAL1) and B4GALT1 increased PG9 sensitivity ~30-fold (Figs 2A and 3). In stark contrast, co-transfection of β-galactoside α-2,3-sialyltransferase 4 (ST3GAL4) markedly *reduced* PG9 sensitivity (Fig 2A), suggesting a preference for α-2,6 SA termini [4, 36, 39, 43].

Overexpression of N-acetylneuraminide α-2,8-sialyltransferase 4 (ST8SIA4), which transfers additional SA moieties onto SA termini (Fig 1), also increased PG9 sensitivity (Figs 2A and 3). However, the increase was not as strong as with the B4GALT1-modification alone. Therefore, we suggest that ST8SIA4 (used with B4GALT1) in fact has a mild inhibitory effect. Finally, the removal of both α-2,3- and α-2,6-linked SA termini by NA reduced PG9 sensitivity by >5-fold, further emphasizing the role of SA contacts.

Gp120-gp41 interface bnAbs were dramatically affected by late GE (Fig 2A). Since PGT151 recognizes tri- and tetra-antennary glycans [3, 4, 61] and is adversely affected by kifunensine, swainsonine, GNT1- and 2FF [78], we were surprised that B4GALT1+/-ST6GAL1, ST3GAL4 or ST8SIA4 also decreased PGT151 sensitivity (Figs 2A and 3). 2FG had a mild inhibitory effect, suggesting galactose-dependency. Conversely NA had no impact, suggesting impartiality to SA termini [3, 4, 61].

In stark contrast, the same late GE methods dramatically increased 35O22's potency by >100-fold. This was paradoxical, considering the similar enhancing effects of kifunensine, GNT1- and 2FF (Figs 2A and 3). On the other hand, the slight loss of sensitivity with NA treatment is consistent with previous reports that 35O22 recognizes complex glycans (N88, N241 and N625 in the JR-FL strain) [4, 21, 23]. Later, we address the unexpected effects of late modifications on PGT151 and 35O22 sensitivities.

BnAbs VRC34 and 3BC176 were more modestly affected by late GE, although the spread of effects was somewhat wider for 3BC176, with B4GALT1+ST3GAL4-modified PV being the most sensitive (Figs 2A and 3). B4GALT1+/-ST6GAL1 and NA were found to increase PGT121 sensitivity (S2 Fig). Otherwise late GE generally only moderately affected V3 glycan, CD4bs and MPER bnAbs. However, NA increased 2F5 resistance, perhaps because removing negatively charged SA reduces electrostatic repulsion with the membrane, so that the trimer sits deeper in the membrane.

### BN-PAGE reveals GE-induced trimer changes

**Fig 4:**
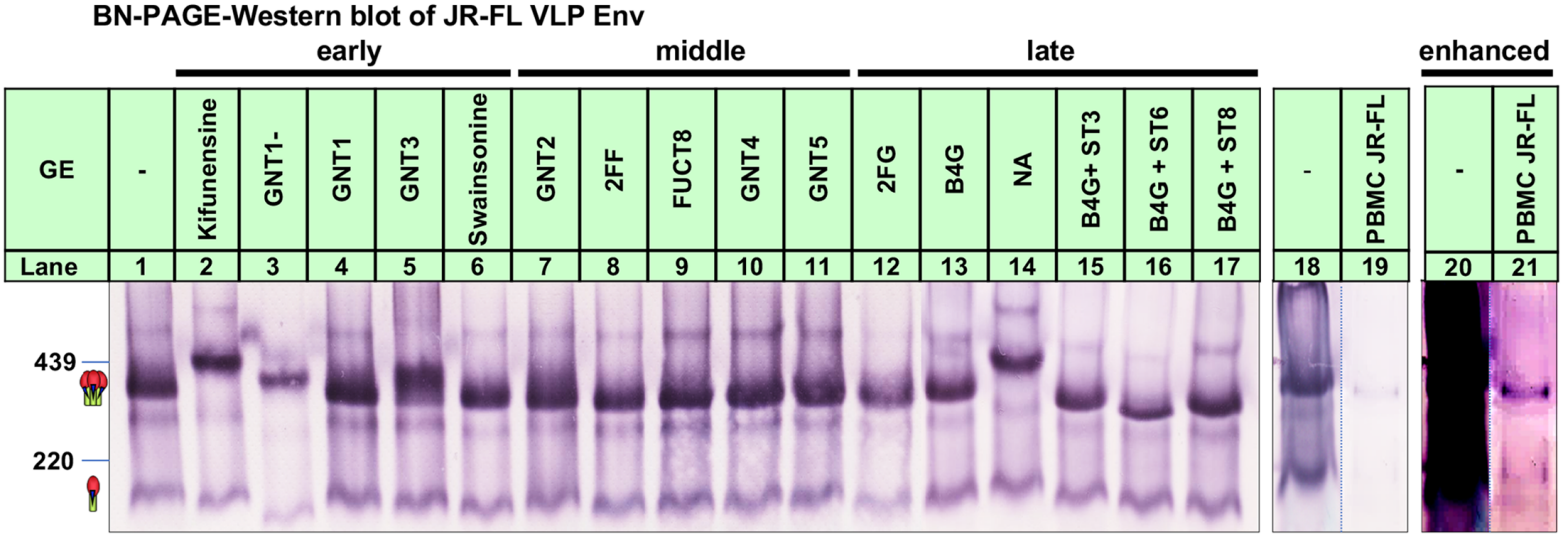
BN-PAGE-Western blot analysis of the effects of GE on particulate Env. Equal volumes (500x concentration) of GE-modified JR-FL SOS E168K gp160ΔCT VLPs (produced using MuLV Gag and Rev) and human PBMC-propagated replicating JR-FL virus were lysed and analyzed by BN PAGE-Western blot. Env was detected using an anti-HIV primary cocktail (39F, 4E10 and 2F5). Trimer and monomer bands are indicated by cartoons along with ferritin markers. Lanes 20 and 21 are enhanced versions of lanes 18 and 19 to better visualize PBMC Env (lane 21). Abbreviations: B4G = B4GALT1, ST6 = ST6GAL1, ST3 = ST3GAL4, ST8 = ST8SIA4.

To contextualize the above findings, we extracted Env from GE-modified virus-like particles (VLPs) and ran them in blue native PAGE (BN-PAGE)-Western blot. Previously, we showed that Env resolves into two bands in BN-PAGE: trimers, largely consisting of functional gp120/gp41, and monomers, largely consisting of high-mannose uncleaved gp160ΔCT [81, 82] (Fig 4). GE caused some marked changes. Kifunensine, GNT1-, GNT3 and NA trimers all migrated relatively slowly (Fig 4, compare lanes 1, 2, 3, 5 and 14). This could be due to bulky untrimmed Man_9_GlcNAc_2_ glycans (kifunensine), the added mass of a bisecting glycan (GNT3) and, perhaps most importantly, the lack of negatively charged SAs which assist in trimer migration (kifunensine, GNT1- and NA). Conversely, B4GALT1+ST3GAL4/ST6GAL1/ST8SIA4 trimers all migrated faster than the control (Fig 4, compare lanes 1, 15–17), suggesting that additional, negatively charged SA improves trimer migration. GNT1-, kifunensine, 2FG and B4GALT1+ST6GAL1 trimers were all relatively poorly expressed (Fig 4, compare lanes 1, 2, 3, 12 and 16). The weaker expression GNT1- and kifunensine trimers may account for the low PV infectivities that we observed earlier (S1A Fig).

We also analyzed a concentrated stock of replicating full-length JR-FL virus grown in PBMCs using image enhancement to assist comparison with VLP Env (Fig 4, lanes 18 and 21). The PBMC trimer migrated slightly faster than the VLP trimer. This was unexpected, given that the VLP Env (gp160ΔCT) lacks 148 C-terminal amino acids, resulting in an expected reduction in trimer mass of ~48.9 kDa (~16.3 x 3). We suggest that the relatively fast mobility of PBMC trimer is driven by Env hypersialylation [19].

### SDS-PAGE-Western blot reveals B4GALT1-induced glycan "thinning"

To further contextualize the neutralization analysis above, we analyzed GE-modified VLP gp120 and gp41 in SDS-PAGE-Western blots. Fine details can be found in S1 Text and Figs A and B contained therein. Briefly, the key findings were as follows: i) B4GALT1 increased Env endo H-sensitivity, consistent with the partial replacement of complex glycans with unfucosylated hybrid glycans. This may explain why B4GALT1 unexpectedly reduced PV sensitivity to multiantennary glycan-preferring bnAb PGT151 and increased sensitivity to the small glycan-preferring bnAb 35O22. ii) GNT3, swainsonine and 2FF also increased Env endo H-sensitivity, albeit less effectively than B4GALT1, suggesting that these methods also replace complex glycans with hybrid glycans, albeit less effectively than B4GALT1. iii) Kifunensine and GNT1- Env consisted of relatively homogeneous, endo H-sensitive high mannose glycans, as expected.

Given that B4GALT1+/-ST6GAL1 impacts PG9, 35O22 and PGT151 sensitivities (Figs 2A and 3) and promotes hybrid glycans (S1 Text), we wondered what effect ST6GAL1 alone might have. Unlike B4GALT1, ST6GAL1 alone did not affect gp120 or gp41 endo H sensitivity, suggesting that it does not promote hybrid glycans (S5A Fig). However, it nevertheless increased PG9 sensitivity to near equivalent levels as B4GALT1+ST6GAL1 PV (S5B Fig). This suggests that cellular galactosylation is sufficient for ST6GAL1 to attach α-2,6 SA termini. However, unlike the B4GALT1 treatments, ST6GAL1 alone did not increase 35O22 sensitivity and only modestly decreased PGT151 sensitivity (S5B Fig). As expected, VRC01 was unaffected. Referring to Fig 1, upon the formation of a hybrid intermediate in the medial Golgi, there is a bifurcation in the pathway, where glycans either mature into complex or hybrid glycoforms. We suggest that B4GALT1 overexpression diverts glycoprotein traffic in the latter direction, so that normally complex glycans (magenta in S5C Fig) are replaced by smaller hybrid glycans (orange in S5C Fig), thus "thinning" Env glycan coverage. Co-transfection of ST6GAL1 and B4GALT1 further improves PG9 sensitivity by promoting sialylation (yellow in S5C Fig). According to Fig 1, swainsonine, 2FF and GNT3 modifications might also divert traffic to the hybrid glycan branch. However, judging from gp41 endo H laddering patterns, B4GALT1 is more effective (S1 Text).

### GE effects on BG505 neutralization sensitivity

To further assess the effects of GE, we next analyzed the vaccine strain BG505 T332N gp160ΔCT (referred to as 'BG505' hereafter), focusing on the GE tools that markedly impacted JR-FL sensitivity: GNT3, 2FF, swainsonine, B4GALT1, ST3GAL4, ST6GAL1 and NA. Kifunensine and GNT1- PV infection was undetectable (S1B Fig). Several other treatments also reduced infection more significantly than they did for JR-FL (S1A and S1B Fig). The effects of GE on BG505 bnAb sensitivity are shown in Fig 2B and S6 Fig. As for JR-FL, BG505 resistance to 14e or F105 was unaffected by GE (S6 Fig). Also, as for JR-FL, PG9 potency was improved against B4GALT1+ST6GAL1 and swainsonine PVs but was reduced against NA PV. In contrast to JR-FL, however, B4GALT1 PV did not show increased PG9 sensitivity and B4GALT1+ST3GAL4 PV did not reduce PG9 sensitivity. Remarkably, B4GALT1+ST6GAL1 increased CAP256.09 sensitivity by ~30-fold and NA decreased sensitivity by a similar factor, consistent with evidence that CAP256.09 contacts terminal SA [4, 36, 40]. As for PG9, B4GALT1 alone had no effect on CAP256.09, whereas the B4GALT1+ST3GAL4 PV was moderately resistant. PGT145, CH01 and VRC38.01 were largely unaffected by GE, except that 2FF inhibited CH01 (S6 Fig), contrasting sharply with its impact on JR-FL sensitivity (Fig 3), suggesting strain-specific GE effects.

As for the JR-FL strain, BG505 sensitivities to V3-glycan and CD4bs bnAbs were largely unaffected by GE (Fig 2B, S6 Fig). However, VRC13 was a notable exception. Unlike most CD4bs mAbs, VRC13 binds to mono- and biantennary glycans bearing terminal galactose in glycan arrays [4]. Contrasting with the mild effects observed for JR-FL, VRC13 neutralization of the BG505 strain was markedly impacted by GE. NA PV was the most sensitive, followed by the B4GALT1 PV. Conversely, swainsonine and GNT3 PVs were less sensitive.

Unlike JR-FL, B4GALT1 overexpression did not markedly affect BG505 sensitivity to PGT151 or 35O22 (S6 Fig), mirroring the similar lack of impact of B4GALT1 on V2 mAbs (also unlike JR-FL). NA slightly enhanced PGT151 sensitivity (S6 Fig)—also not observed for JR-FL (Fig 2). In contrast, B4GALT1+ST6GAL1 mildly inhibited PGT151, suggesting a negative impact of terminal SA. Swainsonine and, to a lesser extent, GNT3, both reduced PGT151 sensitivity, consistent with the partial replacement of multi-antennary glycans with hybrid glycans [4].

None of the GE modifications greatly impacted VRC34.01 sensitivity (S6 Fig). However, 8ANC195 was hypersensitive to B4GALT1+ST6GAL1 PV and was more resistant to NA PV. This suggests α-2,6-linked SA dependency, as we observed for PG9 and CAP256.09. Finally, several GE modifications increased 2F5 sensitivity (S6 Fig) but had less impact on 4E10 and 10E8. Conversely, NA digestion mildly inhibited 2F5 and 4E10, suggesting that, as for the JR-FL strain, removing negatively charged SA moieties may allow the trimer to sit deeper in the membrane, partially obscuring the MPER.

### Effects of GE on CAP256 plasma sensitivity track with the effects of GE on CAP256.09

We next examined whether GE-mediated changes in HIV+ plasma sensitivity matched those of mAbs isolated from the same donors. Donor CAP256 plasma neutralization is overwhelmingly mediated by V2 apex bnAbs of the CAP256 lineage [35]. Donor N152 plasma neutralization is largely mediated by the 10E8 bnAb lineage but is also the source of bnAb 35O22 that only modestly contributes to plasma neutralization [60]. B4GALT1+ST6GAL1 increased the sensitivity of BG505 PV to the CAP256 plasma by 100-fold, mirroring the 30-fold increase in CAP256.09 sensitivity (S7A Fig). However, a BG505 K169E mutant was resistant to both, consistent with the criticial role of the K169 contact (S7A Fig). In contrast, B4GALT1+ST6GAL1 modified JR-FL PV showed modestly improved sensitivity to the N152 plasma, as observed for 10E8, but unlike its dramatic effect on 35O22 sensitivity (S7B and S2 Figs and Fig 3). Thus, the effects of GE on HIV+ plasmas appear to track with predominant bnAb specificities.

### NA reverses ST6GAL1-enhancement of CAP256.09, PG9 and 8ANC195

To formally check that ST6GAL1-mediated increases in sensitivity to PG9, CAP256.09 and 8ANC195 were due to higher numbers of α-2,6 SA termini, B4GALT1+/-ST6GAL1 PVs were subsequently treated with NA and then re-tested for sensitivity to these bnAbs. NA effectively reversed the effects of B4GALT1+ST6GAL1, confirming the importance of SA for these mAbs (S8 Fig). Nevertheless, B4GALT1+NA and B4GALT1+ST6GAL1+NA PVs were not as sensitive as PV treated with NA alone.

In contrast, the effects of the same treatments on JR-FL sensitivity to 35O22 was permanent, i.e. it was not reversed by NA (S8A Fig), confirming that SA was not involved in the gain of 35O22 sensitivity with B4GALT1. PGT151 activity of B4GALT1+/-ST6GAL1 PV was also not fully recovered by NA, again suggesting a permanent effect, although there was a modest gain of sensitivity for B4GALT1 PV (S8A Fig). As noted above, B4GALT1 appeared to have a relatively modest effect on BG505 sensitivity to PGT151 (compare S8A and S8B Fig)—a point we return to below.

### Effects of GE on other gp120/gp41 interface bnAbs and V2 bnAb lineage members

We next examined the effects of GE on two newly reported interface bnAbs ACS202 and CAP248-2B [65, 69]. Details are given in S2 Text and Figs C and D contained therein. In brief, both nAbs were only modestly affected by GE. B4GALT1 reduced ACS202 sensitivity slightly while slightly increasing CAP248-2B sensitivity, suggesting that smaller glycans may resolve clashes in the latter case. Overall, this is consistent with the diverse binding mechanisms of interface bnAbs, where some are markedly affected by GE in different ways (PGT151, 35O22, 8ANC195), whereas others are marginally affected (VRC34.01, ACS202 and CAP248-2B).

We also examined the effects of GE on PG9 and PGT145 developmental relatives. Details are given in S2 Text and Fig E contained therein). In brief, glycan proclivities appeared to be consistent within the mature branches of these lineages.

### Poly-SA termini do not improve V2 apex or interface bnAb sensitivities

We next investigated the possibility that negatively charged poly-SA chains might further impact PV sensitivity to V2 apex and interface bnAbs. This may have been overlooked by our use of B4GALT1+ST8SIA4 above (Figs 2A and 3), as this lacked ST6GAL1 that might be needed to create α-2,6 SA termini as substrates for ST8SIA4 (Fig 1). Details are provided in S2 Text and Fig F contained therein. In brief, poly SA chains formed by B4GALT1+ST6GAL1+ST8SIA4 triple transfection did not accentuate the effects already observed with B4GALT1+ST6GAL1 or B4GALT1+ST8SIA4 component double treatments, suggesting that extra charge does not improve bnAb binding.

### B4GALT1 overexpression consistently promotes hybrid gp41 glycans in diverse strains

Given the differing impact of B4GALT1 on JR-FL and BG505 sensitivities to interface bnAbs 35O22 and PGT151 (Figs 2 and 3, S2 and S6 Figs), we wondered if B4GALT1-induced endo H-sensitivity observed for JR-FL might occur with B4GALT1 treatment of other strains (S1 Text, S5 Fig). Fourteen VLPs from various clades were produced with or without B4GALT1, then analyzed by SDS-PAGE-Western blot and probed for gp41. The gp41 bands of untreated VLPs were, in most cases, largely endo H-resistant, whereas their B4GALT1-modified counterparts were endo H-sensitive, suggesting that it inhibits gp41 glycan maturation (S9 Fig).

The sizes of gp41 bands from strains BG505, JR-FL, WITO and 16055 were relatively small, consistent with their truncated gp41 tails (gp160ΔCT) and staining was also relatively strong. Of these four strains, unexpectedly, BG505 and WITO gp41 bands were partially endo H-sensitive even without B4GALT1 modification (S9A Fig). The complex laddering in both cases suggested substantial glycodiversity. It is possible that the particularly high expression of the BG505 and WITO Env clones leads to these unusual band patterns. Overall, these findings raise the possibility that the milder effects of B4GALT1 on BG505 sensitivities to 35O22 and PGT151 as compared to JR-FL may be because hybrid gp41 glycans are already present in the BG505 strain, thus diluting the impact of B4GALT1 co-expression.

### GE impacts the bnAb sensitivities of diverse strains

**Fig 5:**
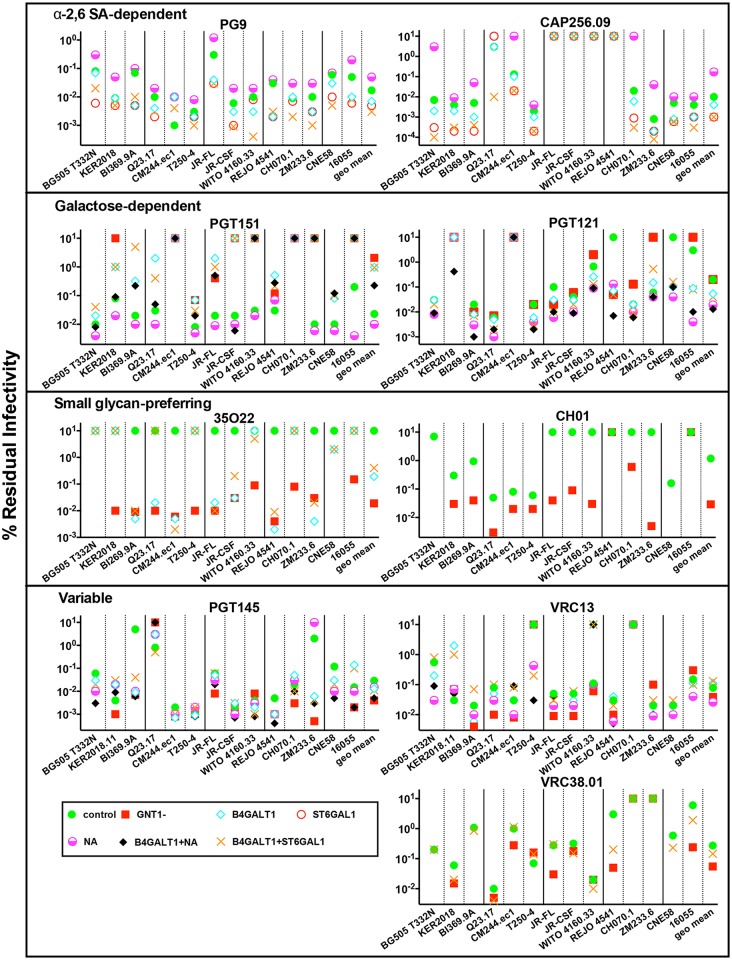
GE effects on the neutralization sensitivities of a diverse panel of virus strains. The effects of various GE modifications on mAb IC50s against a panel of 14 viruses are shown. Particular GE modifications for each nAb were selected as those that markedly affect neutralizing activity against the JR-FL and/or the BG505 strains above. IC50s >10ug/ml were assigned as 10μg/ml. Geometric mean IC50s of all 14 viruses per each GE treatment are shown on the right of each chart, omitting datum for mAb-virus combinations in which IC50s were >10μg/ml under all GE conditions. The infectivities of GNT1- modified BG505 and CNE58 were too low for IC50s to be reliably measured and were therefore omitted. BI369.9A and Q23.17 GNT1- neutralization assays with PGT151 were also omitted due to inconsistent IC50s, in part due to the low infectivity of GNT1- PVs. Results are representative of at least two repeats performed in duplicate. IC50s are shown in S1 Table.

We next investigated the effect of GE on the sensitivities of the same 14 strains (Fig 5). Here, the geometric mean IC50 of each bnAb against all PVs was plotted to the right of each graph. Overlapping dots in Fig 5 are resolved in S1 Table, where IC50s are shown in a heat map. Wilcoxon Signed Rank tests were performed using two columns of data, in which the IC50s of a given mAb against control and GE PVs were paired for each strain (S1 Table). Representative mAb titrations are shown in S10 Fig. Env sequences of these and other strains used in this study are shown in S11 Fig. BnAbs were categorized into 4 groups, as follows:

### α-2,6 SA-dependent bnAbs

In all but one case (CM244.ec1), B4GALT1+ST6GAL1 PVs were more PG9-sensitive. 10 B4GALT1+ST6GAL1 PVs were also more sensitive to CAP256.09. Remarkably, the otherwise CAP256.09-resistant Q23.17 strain was rendered highly sensitive (S10A Fig, S1 Table). Four other PVs: JR-FL, JR-CSF, WITO and REJO were resistant to CAP256.09, due to their lack of the key 169K contact (S11 Fig). B4GALT1 and ST6GAL1 treatments alone also generally increased PV sensitivities to these mAbs. B4GALT1 was, in many cases, less effective for CAP256.09 than for PG9, perhaps because it promotes hybrid glycans that are better tolerated by PG9 [4]. In contrast, NA generally reduced sensitivity to these bnAbs to varying extents. Overall, the improved sensitivity imparted by α-2,6 SA-modification is conserved across multiple clades, and we classify these bnAbs as α-2,6 SA-dependent.

### Terminal galactose-preferring bnAbs

PGT151 sensitivity was consistently, albeit usually modestly, improved by NA, suggesting a conserved preference for terminal galactose [4, 61]. This was particularly marked for the 16055 strain (Fig 5, S10B Fig). Interestingly, GNT1- and B4GALT1 both consistently reduced PGT151 sensitivity, although the extent varied considerably between strains. GE also affected neutralization saturation. Thus, GNT1- modification of the JR-CSF strain completely eliminated PGT151 sensitivity, whereas B4GALT1+/-ST6GAL1 reduced saturation to ~50% (S10B Fig).

For 8 viruses, PGT151 sensitivities of B4GALT1 PVs were increased by subsequent NA treatment (compare B4GALT1 and B4GALT1+NA in Fig 5). However, in all cases except BG505 and JR-CSF, IC50s did not reach the sensitivity achieved by NA alone. Thus, in most cases, B4GALT1 reduces PGT151 sensitivity in a manner that is not fully recoverable by subsequent NA treatment.

PGT121 was also effective against NA PVs and most B4GALT1+NA PVs. In some cases, resistant (or nearly resistant) strains became sensitive with these (and other) treatments (REJO, CNE58, 16055 and KER2018.11), thus increasing PGT121 breadth (Fig 5, S1 Table and S10C Fig). Three of these 4 strains (CNE58 excepted) lack-the canonical N332 glycan, but two have a glycan at position N334 instead (16055 excepted), raising the possibility that GE can help compensate and restore PGT121 binding (S11 Fig). Overall, these findings suggest that PGT121 prefers galactose termini, consistent with glycan array data [4].

### Small glycan-preferring bnAbs

High concentrations of 35O22 did not quite reach an IC50 for all 14 unmodified strains (Fig 5). However, activity was dramatically and consistently increased in 12 GNT1- PVs tested (GNT1- modified BG505 and CNE58 PVs were poorly infectious and were therefore omitted; Fig 5, S1 Table). For 9 strains, B4GALT1 PV also increased 35O22 sensitivity. Notably, B4GALT1+/-ST6GAL1 improved 35O22 saturation of JR-CSF and KER2018.11 PVs, and GNT1- led to a further increase (S10D Fig). This pattern was the exact reverse of that observed for PGT151 (S10B Fig). Since 35O22 binds to complex and high mannose glycans in arrays, we suggest that its greater activity against GNT1- PVs might be due to improved glycan core binding [4]. Overall, GE consistently improved 35O22 neutralization, in part by improving saturation—a point that we return to later below.

Consistent with earlier observations (Fig 3 and S6 Fig), 5 of 5 CH01-sensitive strains became more sensitive with GNT1- modifications. GNT1- modification uncovered CH01 sensitivity in another 5 otherwise resistant strains (Fig 5). In keeping with the lack of CH01 binding to glycan arrays, it appears that smaller Man_5_GlcNAc_2_ glycans consistently minimize clashes. However, two strains (REJO and 16055) remained resistant upon GNT1- modification. Since key CH01 contacts (N156 and N160 glycans and K171) [42] are present, this resistance may be due to remaining glycan clashes.

### Strain-specific effects

Contrasting with the largely consistent and, in some cases, highly significant (S1 Table) patterns above, the most sensitive GE variant for some bnAbs differed between strains. PGT145 potently neutralized many GNT1- PVs but was less effective on Q23.17. NA-treated PVs were also largely sensitive, except for ZM233.6. However, GNT1- and B4GALT1 versions of ZM233.6 were highly PGT145-sensitive (Fig 5; S1 Table). V2 bnAbs are subject to potential clashes with glycans at position N130 and in the V2' region (residues 183–191, stippled pattern in S11 Fig) [45]. None of the 14 strains used in Fig 5 have a N130 glycan, although BG505, BI369.9A and ZM233.6 each have two sequons in the V2' region, while the other strains have one or none (S11 Fig). The dramatically higher PGT145 sensitivities of GNT1- and B4GALT1-modified BI369.9A and ZM233.6 PVs may be because glycan clashes are eliminated. Unmodified BG505 is PGT145-sensitive without GE modifications, suggesting that the V2' glycans of this strain do not limit PGT145 access. The outlier status of the ZM233.6 strain may be related to its unique lack (among these strains) of the N156 glycan (S11 Fig). Overall, PGT145 appears to be glycan-averse and subject to clashes, consistent with its lack of glycan array binding.

GE also had variable effects on VRC13. Half of the GNT1- PVs tested were sensitive. NA also improved sensitivity in most cases. The glycan fence surrounding the primary receptor site [57], typically consists of 4 to 7 glycans, including variable glycans of the V5 loop (S11 Fig). Although VRC13 may clash with some of these glycans, it may contact others. Therefore, since GE may eliminate clashes or modulate mAb-glycan contacts, it is difficult to unequivocally interpret these patterns.

GE also variably affected VRC38.01. Given the lack of glycan array binding, this may be due to glycan clashes. Thus, GNT1- PVs of the REJO and 16055 strains were far more sensitive than control PVs, while the sensitivities of other strains were less affected, and in one case (T250-4), was lower (Fig 5, S1 Table and S10E Fig). The effects of B4GALT1+ST6GAL1 also varied. As mentioned above, all the strains in this panel lack the N130 glycan that clashes with VRC38.01 binding. These strains also exhibit most if not all known VRC38.01 contacts (S11 Fig) [42]. The resistance of the CH070 and ZM233.6 strains may be related to their lack of a tyrosine at position 173 that may help orient the N156 glycan for binding (both strains) and/or the absence of the N156 glycan (ZM233.6 strain; S11 Fig).

### Effects of GE on other nAbs

The activities of other, less broadly neutralizing nAbs were also examined against these 14 strains. Although HJ16 neutralized the 16055 PV and was unaffected by GE [21, 23], it remarkably enhanced infection by Q23.17 and KER2018.11 PVs in various formats (S10F Fig). However, this enhancement was reduced (Q23.17) or eliminated (KER2018.11) against GNT1- versions of these PVs (S10F Fig), implying that HJ16 can activate infection by some strains when they carry complex glycans.

WITO sensitivity to 8ANC195 was knocked in by B4GALT1+ST6GAL1, thereby increasing its breadth (S10G Fig). This effect was partially reversed by NA, as above with the BG505 strain (S8 Fig). Thus, although WITO strain bears the N230 glycan thought to clash with this mAb and lacks the N234 glycan thought to be important for binding, B4GALT1+ST6GAL1 modification was sufficient to allow this mAb to neutralize.

### GE increases neutralization saturation

**Fig 6:**
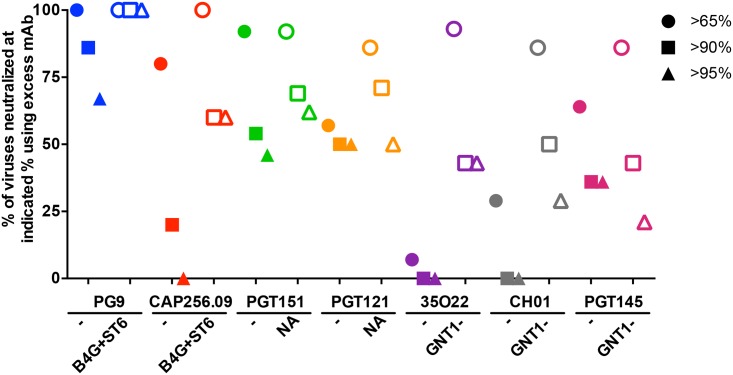
GE improves neutralization saturation. For each mAb, the number of the 14 viruses from Fig 5 that were neutralized above 65%, 90% or 95% in the presence of 10μg/ml of the mAb indicated were expressed as a percentage of the total viruses. Open symbols represent the optimal glycomodification for each nAb; B4GALT1+ST6GAL1 (abbreviated as B4G+ST6) for PG9 and CAP256.09, NA treatment for PGT151 and PGT121, and GNT1- cells for 35O22, CH01 and PGT145. This analysis included data only for those strains that were neutralized in either the control or modified condition (or both) with IC50s <1μg/ml.

In many cases, GE increased the percent maximum neutralization by excess nAb. Perhaps the best examples are 35O22 and CH01 (Fig 2). Using neutralization data from the 14-virus panel (Fig 5), we plotted the % of control and GE-modified PVs neutralized to >65%, >90% and >95% saturation by excess bnAb (10μg/ml). GE dramatically improved saturation by PG9, CAP256.09, 35O22 and CH01 (Fig 6). Notably, PG9 neutralized all B4GALT1+ST6GAL1 PVs to >95% saturation. Although the effects of GE on PGT151 and PGT121 were relatively modest, there was also a positive trend. For PGT145, there was no clear increase in saturation against GNT1- PVs, consistent with the variable effects noted in Fig 5. Overall, despite the small numbers of viruses analyzed here, we infer that, in many cases, GE improves neutralization saturation, in some cases dramatically, either by eliminating glycan clashes and/or by creating optimal glycan structures for optimal binding.

### α-2,6 SA-dependent bnAbs are highly effective against PBMC-passaged HIV-1

Above, we found that certain bnAbs were more effective against B4GALT1+ST6GAL1 PVs. In primary cells, HIV-1 Env is thought to be naturally modified by terminal α-2,6 SA (contrasting α-2,3 SA, as common for 293T cells) [19]. Furthermore, the relatively fast mobility of PBMC-derived trimers in BN-PAGE (Fig 4) suggest possible hypersialylation. We wondered if these factors might make PBMC virus unusually sensitive to these bnAbs. We therefore investigated the change in IC50 of PVs upon B4GALT1+ST6GAL1-modification and the change in IC50 of 293T cell-produced infectious molecular clones (IMCs) upon PBMC passage.

**Fig 7:**
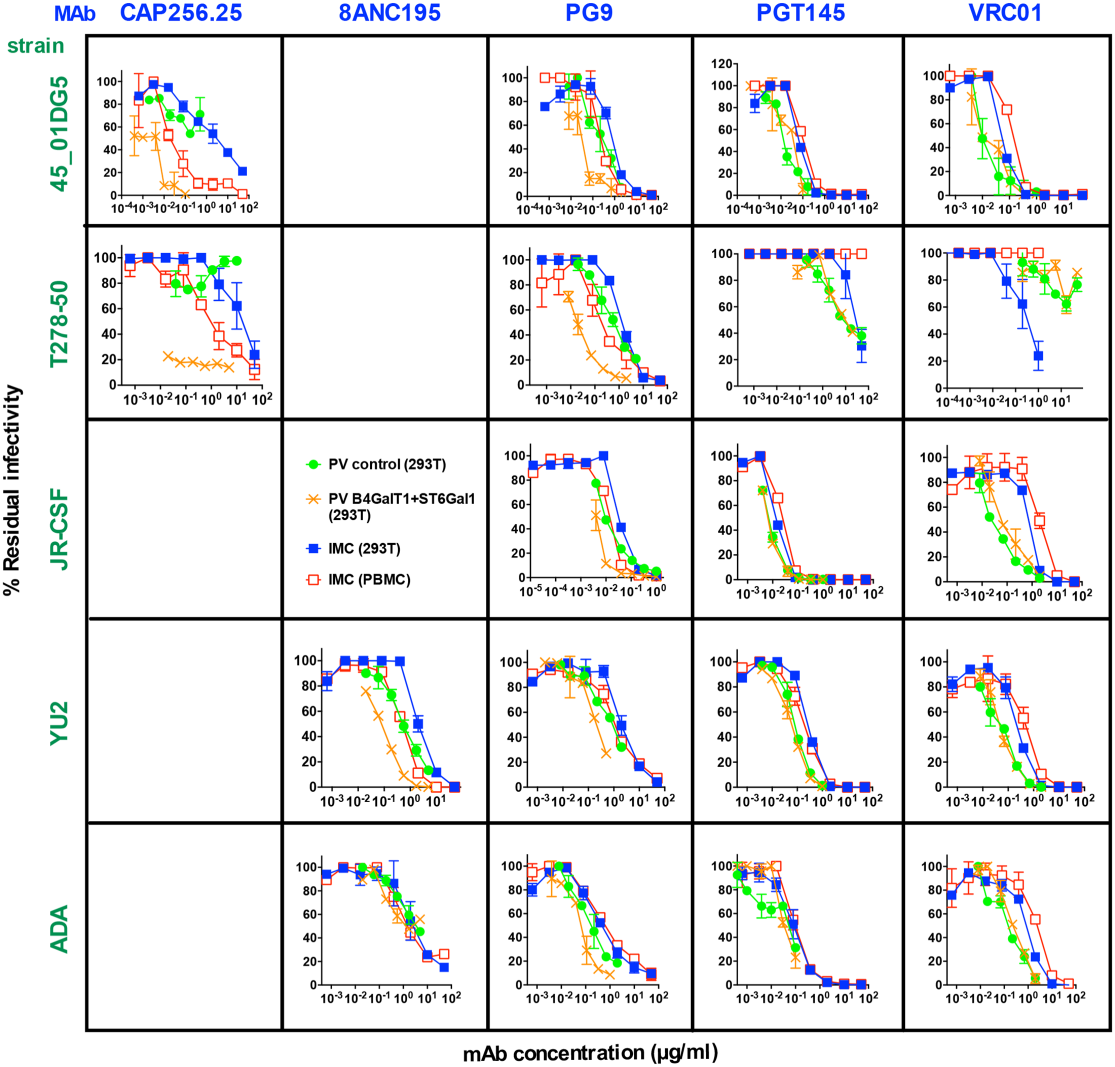
Comparison of the effects of B4GALT1+ST6GAL1 and PBMC passage on PV and IMC sensitivities. Viruses from 5 HIV-1 strains were produced as PVs or infectious molecular clones (IMCs). Some 293T cell-derived PVs were modified by B4GALT1+ST6GAL1, as indicated. Some IMCs were passaged through PBMCs as indicated. Neutralization assays were performed with the addition of indinavir to assays using IMCs to limit infection to a single round. Results are representative of at least two repeats performed in duplicate.

B4GALT1+ST6GAL1-modification of 45_01DG5 and T278-50 PVs rendered them highly sensitive to the α-2,6 SA-dependent bnAb CAP256.25 (Fig 7). Notably, the 45_01DG5 strain bears a methionine at position 165 of the V2 loop C strand (S11 Fig), suggesting that improved glycan contacts may compensate for suboptimal protein contacts. PBMC passage also increased the IMC sensitivity of both strains to CAP256.25 (Fig 7). B4GALT1+ST6GAL1-modification and PBMC passage also improved YU2 sensitivity to 8ANC195. However, ADA sensitivity was unaffected. The sensitivities of 45_01DG5, T278-50 and JR-CSF to PG9 were also increased by B4GALT1+ST6GAL1 modification and PBMC passage. These gains were similar in magnitude to those observed with 8ANC195 on YU2 but less than those with CAP256.25. Although B4GALT1+ST6GAL1-modification marginally improved YU2 and ADA sensitivities to PG9, there was no clear effect of PBMC passage on IMC sensitivity.

In stark contrast to most of the above findings with α-2,6 SA-reactive bnAbs, PGT145 and VRC01 IMC sensitivities were in no case improved by PBMC passage. In fact, sensitivities to VRC01 were reduced in all cases and T278-50 sensitivity was completely knocked out for both bnAbs. Consistent with these findings, B4GALT1+ST6GAL1-modification also did not enhance sensitivity to these mAbs. Overall, these observations suggest that PBMC passage modifies IMCs with α-2,6 SA in a similar way that B4GALT1+ST6GAL1 modifies PV, increasing sensitivities to bnAbs that depend on these hypersialylated termini. However, the sensitivity of PBMC-grown IMCs was, in most cases, lower than that of B4GALT1+ST6GAL1-modified PV.

### BnAb-glycan contacts are not germline-encoded

**Fig 8:**
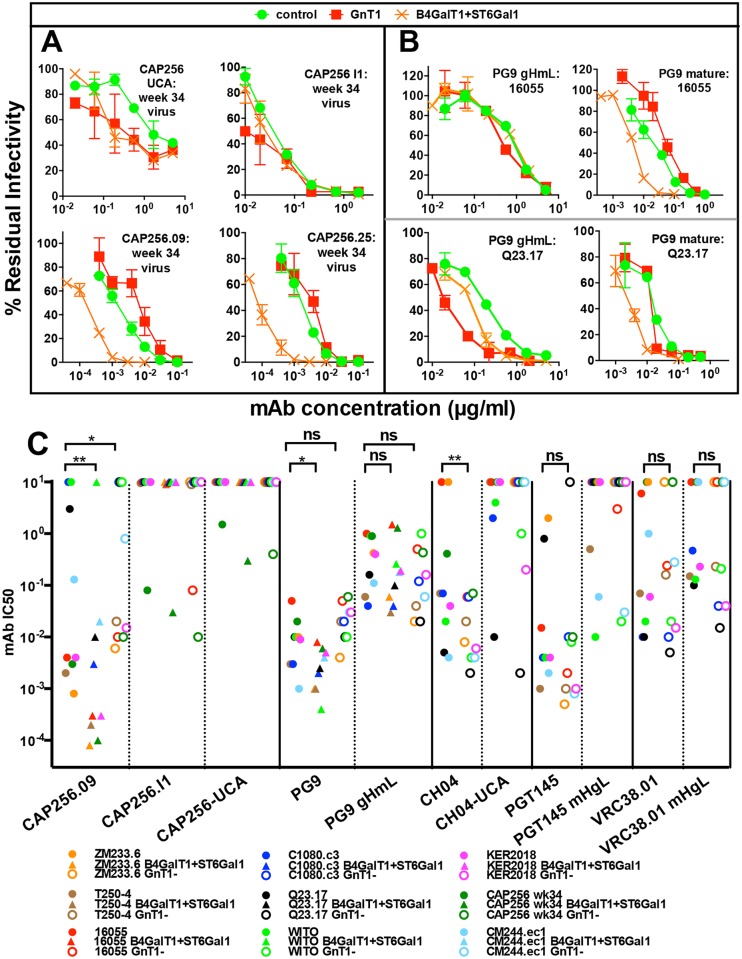
Glycan contacts of mature bnAbs are not germline-encoded. A) GE PVs from donor CAP256 [35] at week 34 were tested for neutralization sensitivity to bnAbs CAP256.09, CAP256.25, intermediate I1 and the inferred unmutated common ancestor (UCA). B) GE-modified 16055 and Q23.17 PVs were tested for sensitivity to PG9 with a reverted heavy chain (gH) mixed with the mature light chain (mL) and fully mature PG9. Results are representative of two replicates; error bars represent SD. C) Nine V2-sensitive strains [34, 36, 45] produced in control, B4GALT1+ST6GAL1 and GNT1- formats were tested for sensitivity to V2 bnAbs and their germline revertants, as indicated. The CH04 UCA (RUA/RUA) was the same as that used in ref [33]. Mixed mHgL versions of PGT145 and VRC38.01 were used that previously showed the most reactivity of the ancestors tested [34, 42]. Results are representative of at least two repeats performed in duplicate. Wilcoxon Signed Rank tests were performed on data for each mAb-PV pair, organized into two columns to compare IC50s for control and GE-modified formats. Significant p values are shown.

Given the key role of glycan contacts for CAP256.09 and PG9, we next investigated their role in sensitivity to germline-reverted versions of these bnAbs. Autologous PV from donor CAP256 sampled at 34 weeks was sensitive to an unmutated common ancestor (UCA) and I1 intermediate CAP256 bnAb and the mature .09 and .25 clones (Fig 8A). However, the effects of GE changed as these Abs matured. The UCA preferentially neutralized the GNT1- and B4GALT1+ST6GAL1 PVs, whereas the I1 intermediate was indifferent to GE and both mature clones showed a strong preference for the B4GALT1+ST6GAL1 PV and weaker neutralization of the GNT1- PV. Together, this suggests that α-2,6 SA binding is not germline-encoded but develops during ontogeny. The stronger GNT1- PV sensitivity to the UCA suggests a benefit of reducing glycan clashes. The higher sensitivity of the B4GALT1+ST6GAL1 PV to the UCA is consistent with B4GALT1-induced glycan "thinning" (replacing complex glycans with hybrid glycans) that may also reduce clashes.

To approximate the PG9 ancestor, we used a germline-reverted heavy chain (gH) co-expressed with the mature light chain (mL) [34, 42]. Since autologous viruses were unavailable, we examined heterologous tier 2 strains. GE did not affect the activity of the revertant against the 16055 PV (Fig 8B). However, Q23.17 GNT1- PV and, to a lesser extent, B4GALT1+ST6GAL1-modified Q23.17 PV were relatively sensitive (Fig 8B). In contrast, the B4GALT1+ST6GAL1 PV of both strains was most sensitive to mature PG9. Overall, this suggests that the SA binding is not a feature of CAP256 or PG9 mAb ancestors, where smaller glycans may be more important to minimize clashes, at least in some settings.

To further investigate the impact of GE on the activities of bnAb ancestors, we used a panel of 9 V2 nAb-sensitive strains [34, 36, 40, 42, 45] that all lack the N130 glycan and have short, sparsely glycosylated V2' regions (S11 Fig). Additional data for the JR-FL strain is shown in S12 Fig. As we observed above (Fig 5), CAP256.09 preferentially neutralized B4GALT1+ST6GAL1 PVs, but the UCA and I1 variants preferentially neutralized the GNT1- and B4GALT1+ST6GAL1-modified autologous PVs (Fig 8A and 8C, S2 Table). The I1 intermediate also neutralized GNT1- modified 16055 strain and (marginally) the T250-4 strain, but no others. Similarly, the PG9 revertant did not exhibit the B4GALT1+ST6GAL1 preference of its mature counterpart (Fig 8B and 8C). For some strains, GNT1- PVs were slightly more sensitive, although this difference was not statistically significant when all 9 strains were considered (Fig 8C, S2 Table).

The high sensitivities of many GNT1- PVs to mature CH04 (a clonal variant of CH01) were mirrored by high sensitivities of GNT1- PVs of some strains to its UCA (WITO, KER2018 and Q23.17; Fig 8C). The effects of GNT1- on PGT145 mHgL revertant sensitivity were mixed, but generally matched those of mature PGT145. For example, the revertant neutralized the KER2018.11 and JR-FL GNT1- PVs more effectively, as did its mature counterpart, although revertant neutralization did not reach an IC50 titer against KER2018.11 (S12A Fig).

KER2018.11, C1080, Q23.17 and JR-FL GNT1- PVs were more sensitive to the VRC38.01 revertant (Fig 8C, S12B Fig). Similarly, a mixed chain VRC13 mHgL revertant neutralized the JR-FL GNT1- PV more effectively, as did its mature counterpart (S12C Fig). Overall, the sensitivities of GNT1- PVs to PGT145, VRC38.01 and VRC13 varied between strains, generally in concert with their mature counterparts, suggesting that strain-specific clashes are important throughout their development and may, in some cases, be alleviated by PV production in GNT1- cells.

Finally, we examined the effects of GE on JR-FL sensitivity to a somatic ancestor and a revertant of PGT121. Neutralization by the 3H3L ancestor derived from deep sequencing [83] was not appreciably enhanced by NA, unlike mature PGT121 (S12D Fig). Moreover, it was most effective against the GNT1- PV, contrasting sharply with mature PGT121. Reverted forms of PGT121, including CDR3mat [84, 85] all failed to neutralize (S12D Fig).

### GNT1- trimers exhibit a tier 2 phenotype suitable for vaccine priming

**Fig 9:**
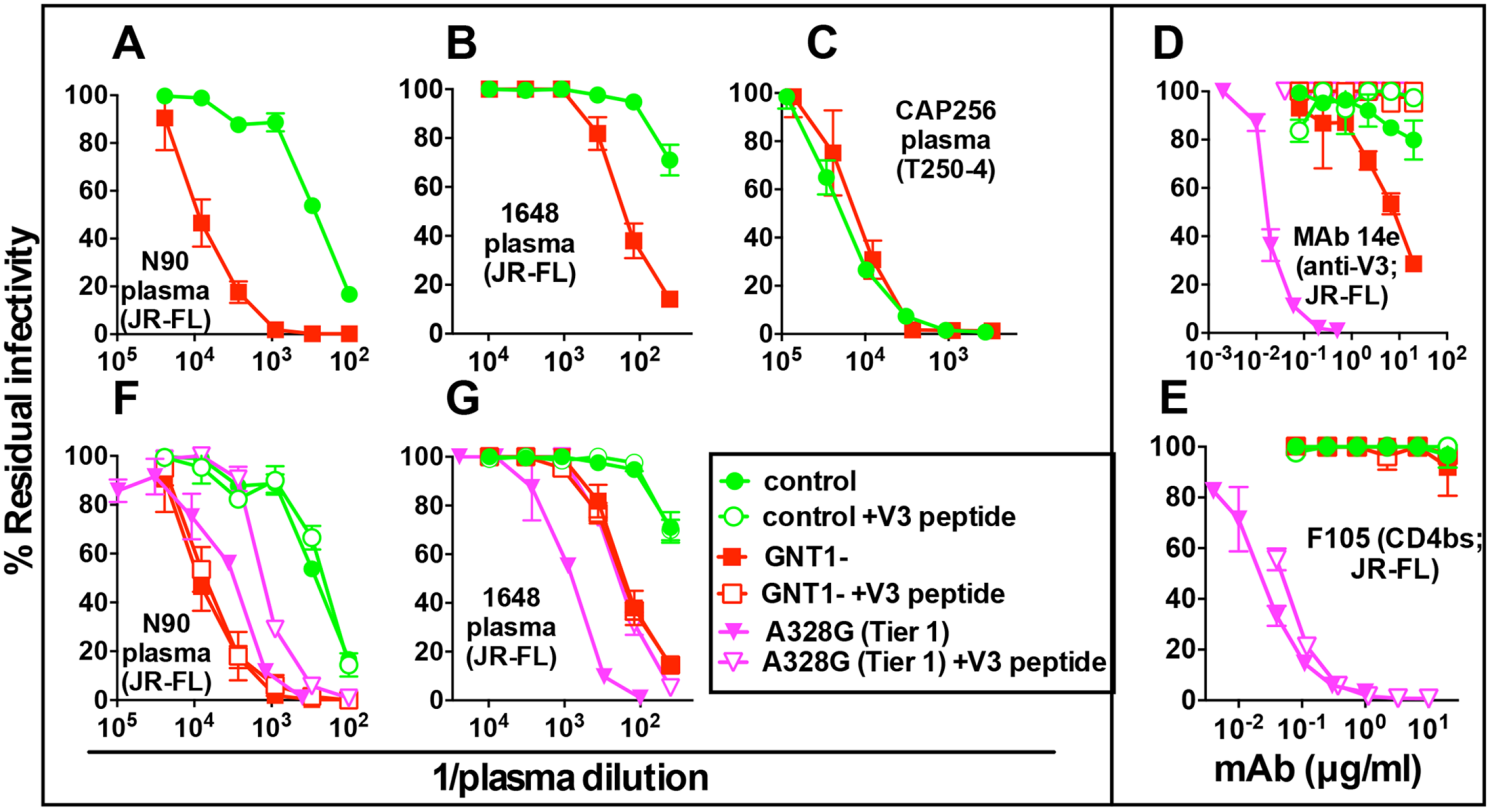
GNT1- PV exhibits a largely V3-resistant tier 2 phenotype. A-C) Comparison of neutralization sensitivities of JR-FL WT control and GNT1- PVs to 3 HIV+ donor plasmas. D-G). The neutralization sensitivity of a V3-sensitive tier 1-like A328G mutant of JR-FL WT was compared to the corresponding GNT1- and control PVs. Assays were performed with or without added JR-FL V3 peptides (30μg/ml). All assays were performed in CF2 cells and used the JR-FL PV except for CAP256 plasma that used T250-4.

The increased sensitivity of GNT1- trimers to germline-reverted bnAbs raises the possibility that they could be useful as vaccine priming immunogens. To be effective in a vaccine context, it may be important to ascertain that GNT1- trimers are compact and V3-resistant like their unmodified counterparts. This would assuage any concerns of 'off target' responses that could drain focus from desired targets. We investigated this question using HIV-1+ donor plasmas. The N90 plasma (source of the VRC38 nAb lineage [42]) neutralized GNT1- JR-FL PV ~30-fold more effectively than the control (Fig 9A). The weakly neutralizing 1648 plasma exhibited a similar increase [86] (Fig 9B). In contrast, the CAP256 plasma neutralized the T250-4 control and GNT1- PVs equivalently [35] (Fig 9C). These differences could reflect the differing glycan proclivities of the bnAbs they contain. However, the high potencies of the N90 and 1648 plasmas against GNT1- PV could also be due to the increased sensitivity of the modified PVs to V3-directed non-nAbs. To investigate, we used peptides to adsorb V3 non-nAbs and a JR-FL A328G mutant which is known to have an overtly V3-sensitive tier 1 phenotype as a reference [79]. V3 mAb 14e neutralized the GNT1- PV somewhat more effectively than the control, whereas the A328G tier 1 mutant was highly sensitive (Fig 9D). Non-nAb F105 (CD4bs) also potently neutralized the A328G mutant, but not the GNT1- PV (Fig 9E). The A328G mutant was also sensitive to the N90 plasma, but slightly less so than the GNT1- PV (Fig 9F). Conversely, the A328G PV was more sensitive to the 1648 plasma than the GNT1- PV, suggesting that V3 neutralization dominates when tier 2 nAb titers are weak (Fig 9G).

Added V3 peptides fully adsorbed 14e but not F105 activity, as expected (Fig 9D and 9E). They also adsorbed A328G neutralization by the N90 and 1648 plasmas. In stark contrast, however, the sensitivities of GNT1- and control PVs were both unaffected (Fig 9F and 9G). This is important, as it implies that the increased sensitivity of GNT1- modified JR-FL PV to the N90 plasma is largely due to increased sensitivity to tier 2 bnAbs, rather than to increased sensitivity to V3 non-nAbs. Quantitatively, if the IC50 of 14e against GNT1- PV of ~10μg/ml (Fig 9D) and infected plasmas contain an estimated ~100–1000μg/ml total of anti-gp120 Abs, the maximum plasma ID50 of 14e-like Abs against the GNT1- PV could be 1:100. This suggests that the increased N90 plasma ID50 (~1:10,000) against the GNT1- PV (Fig 8A) is not due to V3 non-nAbs, as also confirmed by the lack of effect of interfering V3 peptide. In contrast, the ~5-fold reduced A328G mutant sensitivity to the N90 plasma with added V3 peptides suggests that V3 Abs contribute a sizeable fraction of the activity against this tier 1 virus (Fig 9F). Overall, these findings suggest that GNT1- trimers retain a largely V3-resistant, compact tier 2 conformation but can be more sensitive to tier 2 bnAbs and their revertants, making them attractive for use in vaccine priming.

## Discussion

Here we sought to better understand the glycan structures recognized by bnAbs and how this glycoreactivity evolves and might be applied to vaccine design. Our findings suggest that bnAb precursors initially avoid glycans. UCA binding to native trimers may be facilitated when glycan sequons are unusually absent or skipped over (i.e. "glycan holes") [79, 87, 88] or when glycan maturation is stunted, minimizing clashes. Efforts to understand the early events in bnAb development are complicated by several factors. First, in many cases, "UCA-triggering" Env strains are unknown, as are the glycans they carry and, indeed, their form (e.g. gp120/gp41 trimer, gp160 or gp120 monomer). Second, many bnAb ancestors are mere approximations [34], raising questions about how well they represent the behavior of genuine ancestors. Third, UCAs typically exhibit little, if any, neutralizing activity against the triggering viruses, suggesting that more sensitive assays may be needed. *In vitro* trimer binding assays may be more sensitive and informative. In a recent study, the PCT64 UCA did not neutralize autologous virus, but weakly bound to an autologous GNT1- Env trimer, supporting the preference of bnAb ancestors for minimally glycosylated Env [41]. Similarly, CH04 UCA binding to SOSIP trimers was detected when neutralization was not [34]. Accordingly, we are now investigating the binding of bnAb ancestors to GE-modified VLPs by ELISA. Even these assays may be too stringent: evidence of inferred bnAb ancestor triggering even when they fail to detectably bind to the antigen *in vitro* attests to the exquisite sensitivity of the earliest stages of bnAb maturation [84, 85, 88].

During maturation, some nAbs acquire an ability to bind glycans. Indeed, the paratope electropositivity of VRC01 class and some V2 bnAbs increases with maturation [34, 35, 42, 89], ostensibly to facilitate interactions with heavily glycosylated HIV-1 Env spikes that often bear negatively charged SA termini. For VRC01-class bnAbs, this may help accommodate the N276 glycan [21]. For CAP256 bnAbs, α-2,6 hypersialylated trimers may promote the development of SA contacts. In contrast, for some nAbs such as CH01 and 35O22, the preference for small glycans does not change and clashes apparently remain unresolved. PGT151 and PGT121 both preferred NA-treated PV. Electrostatic incompatibility with SA does not explain this behavior, as these mAbs recognize complex glycans with galactose termini [4, 37, 48, 50, 54] and do not preferentially neutralize GNT1- PV.

Overall, there was a remarkable agreement between bnAb GE preferences and their reactivity in glycan arrays [4], with some exceptions: despite the SA-dependency of 8ANC195 observed here, this nAb did not bind in glycan arrays [4]. Similarly, although 35O22 bound complex glycans with SA antennae in arrays, it nevertheless neutralized GNT1- PV highly effectively, perhaps suggesting contact with glycan cores.

Our observation that PG9, CAP256.09 and 8ANC195 "punched above their weight" against PBMC-passaged virus is consistent with an earlier report in which PG9 outperformed b12, 4E10, 2F5 and VRC01 [90]. This, coupled with fast mobility of PBMC trimers in BN-PAGE and similar increased potency of these bnAbs against B4GALT1+ST6GAL1-modified PV all lead to the same conclusion that these bnAbs are more effective against α-2,6 hypersialylated trimers. This has two consequences for vaccine development. First, that these bnAbs may be particularly effective in a clinical setting. Second, that α-2,6 hypersialylated trimers may be ideal for boosting as they better match PBMC virus and may promote the development of α-2,6 SA-dependent bnAbs or else provide a "closer to real life" glycosylation profile to enable other bnAbs to navigate past them.

Another explanation for the outperformance of these mAbs against PBMC-passaged virus could be increased V2 tyrosine sulfation at positions Y173 and Y177, which may increase sensitivity to trimer-preferring bnAbs [91]. However, the lack of increased PGT145 potency with PBMC-passaging suggests that α-2,6 SA modification has a more decisive role in regulating bnAb sensitivity. Sulfation may, however, contribute to the generally higher nAb resistance of PBMC viruses [90, 92, 93]. Indeed, the fast migration of PBMC trimers in BN-PAGE could be due to increased sialylation and/or sulfation.

Our findings raise the question of what factors are important for bnAb development in natural infection. Clearly, the answer may be multi-factorial and may include virus sequence diversity and host antibody repertoire differences. Host-encoded glycovariation may also be a factor. Thus, it may be that the donors who developed CAP256 and PG9 lineages impart α-2,6 SA termini with relatively high efficiency. Conversely, PGT121 and PGT151 may have developed in donors where sialylation was inefficient. 35O22 and CH01 may have developed against viral strains with glycan holes that eliminate clashes or else the donor glycosylation machinery might naturally express Env trimers bearing smaller glycans. Indeed, PBMC-based HIV-1 neutralization assays are notoriously subject to significant inter-donor variability that could in part stem from host glycosylation differences that could toggle bnAb epitope exposure on the Env trimers they express.

**Fig 10:**
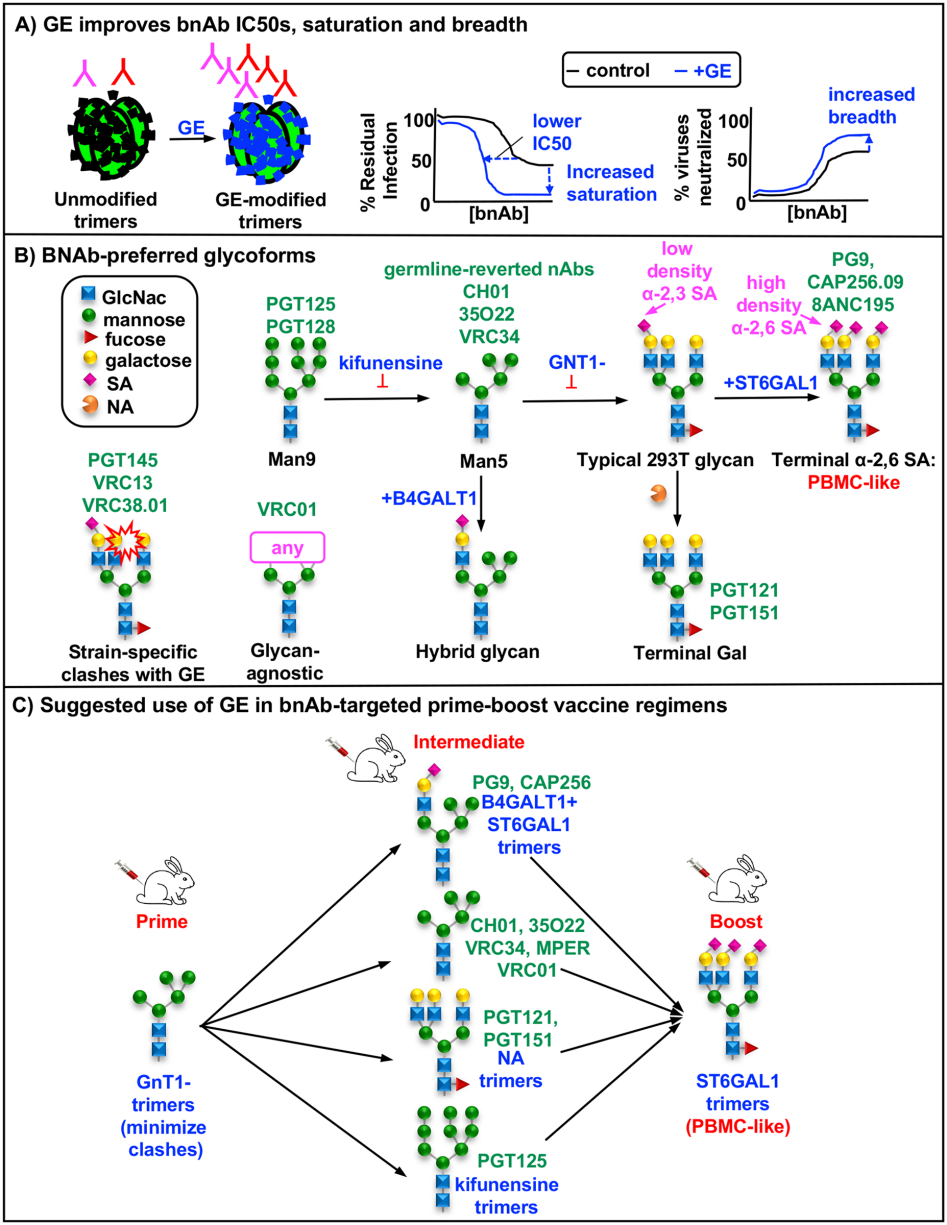
Summary of the effects of GE on bnAb-trimer binding and implications for vaccine development. A) GE increases bnAb sensitivity by lowering IC50 titers, increasing maximum % saturation and increasing breadth. B) The preferred glycoforms of various bnAbs are shown. Preferences include untrimmed high mannose glycans (e.g. PGT125), small glycans (e.g. germline reverted Abs), terminal galactose (e.g. PGT121) and terminal α-2,6 SA (e.g. PG9). Some bnAbs (e.g. VRC01) are largely "glycan agnostic" as they bind protein sites with essentially no glycan clashes that are unaffected by any GE. Other bnAbs (e.g. PGT145) were subject to glycan clashes that varied between strains with GE. PBMC-grown virus trimers are heavily α-2,6 sialylated and best resembles ST6GAL1-modified PV trimers, contrasting with unmodified 293T cell produced PV trimers bear largely α-2,3 SA. B4GALT1 overexpression replaces complex glycans with hybrid glycans. C) GE might be leveraged for use in bnAb-targeted prime-boost vaccine studies. Priming might best use trimers bearing small glycans to minimize clashes with glycan-fearing UCAs. Intermediate immunogens might use trimers bearing GE-modified glycans that are optimal for the bnAb(s) being targeted by this regime. Finally, boosting might best be done using trimers modified with glycans that best resemble those of PBMC-produced viral trimers.

Our key messages are summarized in Fig 10. GE was able to increase bnAb potency, saturation and breadth (Fig 10A), revealing the most sensitive glycoforms for prototype bnAbs (Fig 10B) and suggesting new prime-boost vaccine strategies (Fig 10C). The diffuse gp120 bands observed in Western blots suggest a possible swarm of glycovariants [21, 23]. BnAbs may not be able to neutralize all these variants, providing an avenue for virus 'escape' without mutation. However, GE could resolve this and improve bnAb saturation, either by optimizing glycan contacts and/or by eliminating clashes. Remarkably, several otherwise bnAb-resistant strains were rendered sensitive by GE, uncovering previously unappreciated breadth. Quantifying the extent of this increased breadth will require an analysis of larger numbers of GE-modified PVs.

Our findings provide new opportunities for affinity-based prime-boost native trimer vaccine strategies [84, 85] (Fig 10C). Thus, priming immunogens may be derived from hypersensitive strains that are i) modified to introduce glycan holes at the desired target, ii) mutated to maximize nAb affinity, iii) GE-modified to eliminate glycan clashes and iv) modified to plug glycan holes at unwanted sites. For example, VRC01 priming immunogens might ideally lack the N276 and N463 glycans [7, 28, 94], and be GNT1- modified to further reduce clashes, permitting different angles of approach and improving electrostatic compatibility. In support of GNT1- trimers as priming immunogens, we found that they are recognized poorly by non-nAbs–implying that off-target non-neutralizing responses should be limited. GNT1- modified Q23.17 trimers were hypersensitive to PG9, CH04 and VRC38.01 germline-revertants, raising particular interest in this strain for vaccine development. Although concerns have been raised over its tier 1B classification [95], its resistance to V3 non-nAbs and V2 bnAb hypersensitivity suggest "closed" trimers that may benefit from a naturally short and unglycosylated V2’ region [45, 88].

Intermediate boosting immunogens (Fig 10C) could be the most sensitive GE-modified glycoform for the target bnAb, to encourage the development of glycan contacts. Thus, for example, NA-treated trimers could promote the maturation of PGT121 and PGT151-like bnAbs. Previous work showed that desialylation improves gp120 recognition by some ligands and also improves immunogenicity by reducing electrostatic surface potential [96]. Desialylation may also modify antigen capture and presentation in ways that might promote B cell responses [96]. As mentioned above, the unexpected "glycan thinning" effect of B4GALT1 overexpression (Fig 10B) may be useful for intermediate boosting, to both minimize glycan clashes and also promote SA-reactivity (Fig 10C).

Mass spectrometry analysis of Env glycans may in future allow us to better understand effects of B4GALT1 and other GE-modifications, providing a clearer basis for their effects and their utility in vaccines. In the current study, we were limited to Western blot analyses both by the relatively poor Env yield, and by the co-presence of immature and unprocessed forms of Env that may contaminate trimer glycoanalysis. However, recent improvements in trimer expression and purification methods should facilitate these analyses in the near future [23, 97].

As final boosts (Fig 10C), α-2,6 hypersialylated trimers may be ideal for reasons already mentioned above. The use of CHO or 293T cells for HIV-1 vaccine production may be limited by their tendency to attach α-2,3 SA termini. To address this problem, trimers might be either produced in these cells along with co-expressed ST6GAL1 or could be enzymatically modified after expression to exchange α-2,3 SA with α-2,6 SA termini.

The common development of V2 bnAbs in natural infection, usually with relatively little somatic hypermutation [35, 36, 98] is increasing interest in this site as a vaccine target. Indeed, V2-hypersensitive strain trimers [34, 36, 42] were recently shown to elicit V2 nAbs [45, 88]. Although the long CDRH3 loops of PG9 and CAP256.09 set a high bar for their use as vaccine blueprints, their relatively high potency in PBMC assays raises their significance, especially if other SA-dependent bnAbs with more common-in-repertoire features can be found. By comparison, V2 bnAbs like CH01, VRC38.01, BG1 and PCT64-35S have shorter CDRH3 loops. VRC38.01 may be of particular interest because it is only moderately impacted by glycan variation, thus blocking one avenue of viral escape [39, 43].

In future, GE-modified baits may help to recover additional bnAbs from HIV-1-infected donors to inform vaccine design. GE also provides a way to investigate the effects of glycovariation on the tier 2 nAbs now increasingly being elicited by leading candidate vaccines [57]. This may enable us to develop prime-boost vaccination strategies to elicit broadly protective nAbs.

## Materials and methods

### Ethics statement

All human plasmas were archived (i.e. they were not drawn for this project). Normal (uninfected) human PBMCs with no donor identifiers, sourced from Duke University and NIH blood bank, were used to propagate replicating JR-FL and IMC viruses. Institutional Review Board (IRB) approval for this project was obtained through the San Diego Biomedical Research Institute IRB Committee (approval number: IRB-14-04-JB; Federal Wide Assurance number: 00021327).

### Anti-HIV-1 Env mAb plasmids

MAbs were obtained from their producers and the NIH AIDS Reagent Repository. MAbs included the following (originators given in parentheses): 19b, 39F, F2A3, C011 and 14e (J. Robinson), directed to the gp120 V3 loop [99]; b12 (D. Burton), VRC01 and VRC13 (J. Mascola), 8ANC131 (M. Nussenzweig), HJ16 (A. Lanzavecchia), F105 (M. Posner), directed to epitopes that overlap the CD4bs [67, 99–101]; PGT121, PGT125 and PGT128 (D. Burton) directed to epitopes involving the base of the V3 loop of gp120 and the N332 glycan [37]; VRC38.01, CAP256.09 and CAP256.25 (J. Mascola), PG9, PG16, PGT145 and PGDM1400 (D. Burton), CH01 and CH04 (B. Haynes), directed to V2 apex epitopes [31, 33, 35, 37, 38, 42, 47, 77]; PGT151 (D. Burton), 35O22 (M. Connors), VRC34.01 (J. Mascola), 8ANC195 and 3BC176 (M. Nussenzweig), ACS202 (R. Sanders) and CAP248-2B (P. Moore) directed to the gp120-gp41 interface [60–62, 64, 65, 68, 69]; 4E10 and 2F5 (H. Katinger) and 10E8 (M. Connors), directed to the gp41 MPER [72]. Information on these mAbs can be found at the web link: (www.hiv.lanl.gov). Germline revertants and ancestors were also obtained for mAb lineages CAP256 [35], PG9 [34], PGT145 [34] VRC38 [42], CH04 [33], VRC13 [102] and PGT121 [83–85]. In some cases, variable and J segments were reverted to inferred germline residues, leaving the CDR3 intact. In other cases, UCAs were inferred from nAb ancestors recovered from donors; other ancestors were recovered by deep sequencing.

### Env and Gag plasmids

Plasmid pCAGGS was used to express JR-FL gp160ΔCT on VLP surfaces [99]. Gp160ΔCT is truncated at amino acid 709, leaving a 3 amino acid gp41 cytoplasmic tail. This increases native trimer expression and can be used to produce PVs with similar neutralization sensitivity profiles compared to their full-length gp160 counterparts [99]. Mutants were generated by QuikChange (Agilent Technologies) and were numbered according to the HXB2 reference strain [86]. "SOS" mutations (A501C and T605C) introduce an intermolecular disulfide bond between gp120 and gp41 [99]. The E168K mutation knocks in PG9 epitope and increases trimer expression [99], and the N189A mutation removes a sequon that is competitive with N188, and improves sigmoidal neutralization of V2-targeting nAbs such as PG9.

Plasmids expressing other Env gp160s were obtained from the NIH AIDS repository. Env-deficient sub-genomic plasmid pNL4-3.Luc.R-E- [99], pMuLV Gag (expresses endogenous murine leukemia virus Gag, driven by a CMV promoter) and pMV-Rev 0932 (expresses codon-optimized HIV-1 Rev, driven by a CMV promoter).

We also investigated a series of other Envs, some of which were full-length and others had truncated cytoplasmic tails (gp160ΔCT), as follows. In the following list, clade assignments are given in parentheses. BG505 T332N gp160ΔCT (A), KER2018.11 gp160 (A), BI369.9A gp160 (A), Q23.17 gp160 (A), CM244.ec1 gp160 (AE), T250-4 gp160 (AG), JR-CSF gp160 (B), WITO 4160.33 gp160ΔCT (B), REJO4541 gp160 (B), CH070.1 gp160 (BC), ZM233.6 gp160 (C), CNE58 gp160 (C), 16055–2 clone 3 gp160ΔCT (C), T278-50 gp160 (AG), and 45_01DG5 gp160 (B).

### Glycosyltransferase plasmids

Glycosyltransferase plasmids pEE6.4_B4GALT1 (expresses β-1,4 galactosyltransferase 1), pEE14.4_ST6GAL1 (expresses β-galactoside α-2,6-sialyltransferase 1) and pEE6.4_GNT3 (expresses *N*-acetylglucosaminyltransferase 3) were reported previously [13]. Others were obtained from the DNASU repository: β-1,3-N-acetylglucosaminyltransferases 1–5 (GNT1, 2, 4 and 5), α-1,6-fucosyltransferase (FUCT8), β-galactoside α-2,3-sialyltransferase 4 (ST3GAL4) or α-*N*-acetyl-neuraminide α-2,8-sialyltransferase 4 (ST8SIA4).

### GE

Glycosyltransferase plasmids were co-transfected at a ratio of 1% total transfected DNA. The exception to this was B4GALT1+sialyltransferase (ST3GAL4, ST6GAL1 or ST8SIA4) co-expression, where B4GALT1 was transfected at 1% and the sialyltransferase at 2.5% total transfected DNA. For increasing galactosylation and increasing sialylation, 5 mM D-galactose (Sigma) was added to the medium prior, during and post-transfection in conjunction with co-transfection of B4GALT1. Decoy substrates for blocking fucosylation, 2-deoxy-2-fluoro-l-fucose (2FF) (Carbosynth) and galactosylation, 2-deoxy-2-fluoro-d-galactose (2FG) (Carbosynth), were added 4h post transfection at 0.4 mM and 1mM, respectively. To block the action of mannosidase 1, kifunensine was added at 25 μM during and post transfection. Swainsonine was added at 20 μM during and post transfection to block mannosidase 2 activity. Terminal SAs were cleaved by incubating 25 μl of 1,000x concentrated PV with 2 μl of NA from *C*. *perfringens* (Sigma, Cat# N5631, 5u/ml) and 1 μl of NA from *A*. *ureafaciens* (Roche, Cat # 10269611001, 10U/ml)) for 1 h at 37°C. Both NAs cleave α-2,3, α-2,6 and α-2,8 SA, but *C*. *perfringens NA* preferentially cleaves α-2,3 SA, whereas *A*. *ureafaciens* NA preferentially cleaves α-2,6 SA. PV was then washed with PBS, pelleted and resuspended at 1000 x.

### VLP production

VLPs were produced by co-transfecting human embryonic kidney 293T cells (ATCC) with an Env-expressing plasmid (typically pCAGGS JR-FL gp160ΔCT SOS E168K and mutants thereof), pMuLV Gag and pMV-Rev 0932 using polyethyleneimine (PEI Max, Polysciences, Inc.), as described previously [99]. Two days later, supernatants were collected, precleared by low speed centrifugation, filtered, and pelleted at 50,000 x g in a Sorvall SS34 rotor. To remove residual medium, VLP pellets were diluted with 1ml of PBS, then re-centrifuged at 15,000 rpm and resuspended in PBS at 1,000 x the original concentration.

### Virus production in peripheral blood mononucleocytes

Replicating JR-FL virus was propagated from a stock provided by the NIH AIDS Reagent Repository (CAT#395, donated by Dr Irvin Chen), by cell-free infection of uninfected human peripheral blood mononucleocytes (PBMCs) activated in RPMI medium containing 20% FBS, 50μg/ml gentamycin, 5μg/ml PHA-P (Sigma Cat# L1668) and 5% IL-2 for 12–24 hours followed by washing and resuspension in RPMI supplemented with only 20% FBS and 5% IL-2 for infection. Virus supernatant was inactivated using 1mM aldrithiol and then was concentrated in the same manner as VLPs for use in gel analyses.

### Reference plasmas

HIV-1-infected donor plasmas N90, N152, CAP256, BB34, 1688, 1702, and N160, and uninfected control plasma 210 have all been described previously [35, 60, 72, 86]. These were obtained from the Laboratory of Immunoregulation, NIAID (N90 and N152), The National Institute for Communicable Diseases, Johannesburg, South Africa (CAP256, BB34) and Zeptometrix, Inc., Buffalo, New York, USA (1648, 1688, 1702, N160, 210).

### Blue Native PAGE (BN-PAGE)-Western blots

Blue native polyacrylamide gel electrophoresis (BN-PAGE) was performed as described previously [99]. Briefly, VLPs were solubilized in 0.12% Triton X-100 in 1 mM EDTA. An equal volume of 2x sample buffer (100 mM morpholinepropanesulfonic acid (MOPS), 100 mM Tris HCl, pH 7.7, 40% glycerol, and 0.1% Coomassie blue) was added. Samples were then loaded onto a 4–12% Bis-Tris NuPAGE gel (Invitrogen) and separated at 4°C for 3 hours at 100V. Proteins were then transferred to polyvinylidene difluoride (PVDF) membrane, destained, immersed in blocking buffer (4% nonfat milk in PBST) and probed with an anti-gp120 cocktail (39F, F2A3, C011 and 14e at 1μg/ml) and/or an anti-gp41 cocktail (2F5 and 4E10 at 1μg/ml). Blots were then probed by an anti-human Fc alkaline phosphatase conjugate (Accurate Chemicals) and developed using SigmaFast BCIP/NBT substrate (Sigma).

### SDS-PAGE-Western blots

Env proteins were resolved by reducing SDS-PAGE. Briefly, samples were reduced and denatured by heating at 90°C for 10 min in LDS buffer (Invitrogen) prior to loading onto 4–12% Bis-Tris NuPAGE gels (Invitrogen). SDS-PAGE-Western blots were performed as described above for the BN-PAGE Western blotting method. For the cleavage of oligomannose and hybrid glycans, endonuclease H (endo H) (New England Biolabs) was added to samples after reduction and denaturation, and incubated for 37°C for 1 h prior to SDS-PAGE-Western blotting.

### Neutralization assays

#### PV assays in CF2 cells

Heat-inactivated sera and protein A-purified serum IgGs were analyzed for neutralization of various PVs produced by co-transfecting HEK-293T cells with an Env plasmid and pNL4-3.Luc.R-E- [75]. The neutralization sensitivities of WT and SOS versions of JR-FL are known to be comparable [81]. Neutralization assays using canine CF2 cells expressing CD4 and CCR5 receptors (CF2Th.CD4.CCR5; obtained from Dr. J. Sodroski, Dana Farber Cancer Institute, Harvard University, Boston, Massachusetts, USA) were described previously [81]. Briefly, PV was incubated with graded dilutions of mAb or serum for 1 h at 37°C. The mixture was then added to CF2 cells, incubated at 37°C. For WT PVs, cells were cultured for a further 3 days, then luciferase activity was measured. For SOS mutant PVs, after 2 h, infection was activated by adding 5 mM DTT for 15 min, followed by replacing with fresh medium [81] and culturing at 37°C for a further 3 days, after which luciferase activity was measured. Data shown is representative of at least 2 repeat assays performed in duplicate.

#### PV assays in TZM-bl cells

TZM-bl assays were performed in a similar manner to CF2 assays except that PVs were produced by co-transfecting HEK-293T cells with an Env plasmid and pSGΔ3 plasmid. Antibody-PV samples were incubated together before adding TZM-bl cells (obtained from Dr. David Montefiori, Duke University, Durham, North Carolina, USA) were added, followed by 3 days of culture, after which luciferase activity was measured.

#### IMC assays in TZM-bl cells

Neutralization sensitivities of HIV-1 infectious molecular clones (IMCs) prepared as described previously [93] were evaluated using TZM-bl target cells. IMCs were initially produced from 293T cells by IMC plasmid transfection. The resulting replicating stock was then passaged in PBMCs for 7 to 10 days. PBMCs were isolated from buffy coats of blood draws from the NIH blood bank. In neutralization assays, virus was incubated with mAb for 30 min at 37°C, then added TZM-bl cells. To limit the viral infection to one round, indinavir, a protease inhibitor, was added to the medium. At 48h post-infection, luciferase was assayed.

### Statistics

Wilcoxon Signed Rank tests were performed on data for each mAb-virus pair, organized into two columns to compare IC50s under control and GE-modified conditions.

### Oligomannose arrays

Oligomannose arrays were printed using Man_5_GlcNAc_2_, Man_6_GlcNAc_2_, Man_7_GlcNAc_2_D1, Man_7_GlcNAc_2_D3, Man_8_GlcNAc_2_D1D3 (D denotes the arms bearing terminal mannose groups; see Fig 1), and Man_9_GlcNAc_2_ at 33μM (Z Biotech). GlcNAc_2_ was printed at the same concentration. Print buffer without glycans was included as a background control. Each array was hydrated for 2 min in ultrapure water and then blocked for 1 h with hydrazide glycan blocking buffer (Zbiotech), rotating at 40 rpm in the dark. Arrays were inserted into a SlideArray holder (SlideArray) to partition the array into 24 subarrays. MAbs were diluted to 50 μg/mL in hydrazide glycan assay buffer. PGT128 and biotinylated Concanavalin A were used as positive controls and V3 mAb 19b was used as a negative control. Each mAb was incubated on an individual subarray for 1h and then washed 5 times with PBS/0.05% tween 20 (PBST). Subarrays that received biotinylated Concanavalin A were incubated with streptavidin-Cy3 (Sigma). All other wells were incubated with anti-IgG-Cy3 (Sigma) for 1h while rotating at 40 rpm covered from light. The arrays were washed 5 times with 70μL of PBST and then washed once with 0.01X PBS and then dried. The arrays were scanned with a GenePix 4000B (Molecular Devices) scanner at wavelength 532nm using GenePix Pro7 software. The fluorescence within each feature was background subtracted using the local method in GenePix Pro7 software (Molecular Devices). Glycan specific binding = (glycan binding background-subtracted fluorescence) − (print buffer alone background-subtracted fluorescence).

### Molecular modeling

A JR-FL native Env trimer structure (PDB: 5FUU) [3] was used to model native spike glycans. First, atomic clashes in the 5FUU structure were relieved and missing side-chains were rebuilt by running a constrained ROSETTA-relax simulation. Each sequon was decorated with a Man_9_GlcNAc_2_ glycan. The glycan at position N637 in gp41 is absent, per evidence that one or other glycans at N625 and N637 remain unoccupied [18]. For any overlapping sequons, e.g. those at N188 and 189, only the first sequon is occupied. GlycanRelax [103] was used to approximate glycan conformational behavior. For each model, 10 separate GlycanRelax trajectories of 10,000 cycles of MonteCarlo trials were carried out. Each gp120 glycan could move independently throughout the GlycanRelax minimization. A single low energy model was generated using PyMOL Software (Version 1.5.0.4 Schrödinger, LLC).

## Supporting information

S1 Fig GE effects on PV infectivity. The effects of various GE treatments on A) JR-FL and B) BG505 PV infectivities were measured in CF2.CD4.CCR5 cells. Infectivity is shown relative to the untreated control, set to 100%, with treatments separated into those that affect early, middle and late stages of the N-linked glycosylation pathway. NA treatment involved a 37°C incubation for 1h followed by a PBS wash. The resulting PV and a mock (no enzyme) incubation control both had largely undiminished infectivities as compared to the "no treatment" control. All assays were repeated at least 6 times. Error bars show SD.
(PDF)

S2 Fig Effects of GE on JR-FL sensitivity to V3-glycan, CD4bs and MPER bnAbs. Results are representative of at least two repeats performed in duplicate performed in duplicate. Error bars show standard deviations (SD). IC50s are shown in Fig 2.
(PDF)

S3 Fig VRC38.01 does not bind oligomannose glycan arrays. 33μM of GlcNAc2 and various oligomannose glycans were printed and checked for binding by 50μg/mL of various mAbs and Concanavalin A. Fluorescence was background-subtracted using the local method in GenePix Pro7 software. Means and standard errors of 6 replicates are shown from two independent tests.
(PDF)

S4 Fig Assay-dependent differences in CH01 and 35022 neutralization saturation. The neutralizing activities of mAbs CH01, 35O22 and VRC01 against the JR-FL E168K+N189A PV were compared in the TZM-bl and CF2 assays. All assays were repeated at least 3 times in duplicate. Error bars show SD.
(PDF)

S5 Fig B4GalT1 overexpression restructures gp41 glycans and impacts JR-FL sensitivity to interface bnAbs. The effects of B4GalT1 and ST6Gal1 alone and together on JR-FL Env was assessed. A) Changes in the mobility of JR-FL SOS E168K gp160ΔCT Env produced with the indicated plasmid co-transfections were or were not treated with endo H and then analyzed by SDS-PAGE-Western blot, with or without endo H treatment. Dots indicate Env species, as in Fig A and B of S1 Text. B) Sensitivities of GE-modified JR-FL PVs to mAbs PG9, 35O22, PGT151 and VRC01. Results are representative of two repeats performed in duplicate. Error bars show SD. C) Model of the effects of GE on JR-FL Env trimers. The glycosylated JR-FL 5FUU structure [3] was modeled, with glycans colored coded, as in a previous JR-FL SOSIP trimer analysis [23], to illustrate how B4GalT1 overexpression drives the conversion of complex glycans (magenta) to form hybrid glycans (orange) and that B4GalT1+ST6Gal1 expression drives α-2,6 sialylation of these glycans (yellow). Glycan assignments are for illustrative purposes only.
(PDF)

S6 Fig Effects of GE on BG505 neutralization sensitivity. The effects of modifying selected steps in BG505 T332N WT trimer glycan maturation on mAb sensitivity was assessed in a manner analogous to Fig 3. Kifunensine treatment and GnT1- PV had infectivities too low to be measured reliably and were therefore omitted. Results are representative of two repeats performed in duplicate. Error bars show SD. IC50s are shown in Fig 2B.
(PDF)

S7 Fig Effect of GE on HIV+ plasma neutralization reflects the nAb specificities they contain. A) Sensitivities of control and B4GalT1+ST6Gal1-modified BG505 PV with or without a K169E knockout mutation (to knock out CAP256.09 lineage binding) to plasmas from infected donor CAP256 and CAP256.09, a bnAb isolated from CAP256 donor. B) Comparison of the sensitivities of control and B4GalT1+ST6Gal1 JR-FL PVs to plasma from infected donor N152 and 35O22, a bnAb isolated from N152 donor. Results are representative of two repeat assays performed in duplicate; error bars show SD.
(PDF)

S8 Fig Reversal of B4GalT1+ST6Gal1 treatment by SA cleavage. Changes in sensitivity of glycomodified JR-FL (A) and BG505 PVs (B) after NA digestion were assessed. PV produced with co-transfected B4GalT1 alone and B4GalT1+ST6Gal1 were compared with the untreated control, with or without NA digestion. Results are representative of at least two repeats performed in duplicate. Error bars represent standard deviations.
(PDF)

S9 Fig B4GalT1 co-transfection consistently induces partial gp41 endo H-sensitivity. The effects of B4GalT1 co-transfection on Envs from 14 different strains were compared by SDS-PAGE-Western blot. VLPs expressed A) without or B) with co-transfected B4GalT1 were loaded at equal concentrations without or with endo H treatment and probed with an anti-gp41 primary mAb cocktail (2F5 and 4E10). Dots denote different Env species, as in Fig A and B of S1 Text.
(PDF)

S10 Fig GE-induced changes in bnAb titer, breadth and saturation. MAb titrations against different virus strains under various conditions show some of the titrated effects summarized as IC50s in Fig 5. All assays were repeated at least 2 times in duplicate. Error bars show SD.
(PDF)

S11 Fig Sequence alignment and predicted glycans of strains used in this study. Amino acid sequences were aligned by the TCoffee method using JalView software. Sequons are shaded in yellow. Sequence positions were standardized to the HXB2 strain. The BG505 sequence contains a T332N mutation and the JR-FL sequence contains E168K+N189A mutations, the latter converting NNTS to NATS in the V2' sequence (residues 183–191, shown as stippled).
(PDF)

S12 Fig Ancestors of PGT145, VRC13 and PGT121 preferentially neutralize GnT1- modified virus. A) Sensitivities of control and GnT1- modified KER2018.11 and JR-FL WT PVs to mature PGT145 and its mHgL ancestor; B) Sensitivities of control and GnT1- modified JR-FL WT to mature VRC38.01 and its mHgL ancestor; C) Sensitivities of control and GnT1- modified JR-FL WT to mature VRC13 and its mHgL ancestor; D) Sensitivities of control, NA-treated and GnT1- JR-FL WT PVs to neutralization by mature PGT121 and its ancestors 3H3L and CDR3mat.
(PDF)

S1 Text SDS-PAGE-Western blot reveals B4GalT1-induced glycan "thinning". (PDF)

S2 Text Further investigation of interface bnAbs, V2 bnAb lineage members and poly SA termini. (PDF)

S1 Table Effects of GE on mAb IC50s against a diverse virus panel. MAb neutralization sensitivities of a 14-virus panel was tested using select GE modifications. The most sensitive GE modification for each mAb-strain combination is boxed. Some GE PV infectivities were too low to be measured reliably, denoted as ND (not determined). Geometric means are shown, omitting IC50s >10μg/ml under all GE conditions for a particular mAb-virus combination. Wilcoxon Signed Rank tests were performed on data for each mAb-PV pair organized into two columns to compare IC50s under control and GE conditions. p values showing significant increased or decreased sensitivity are shown by blue or red asterisks, respectively, or were not significant (ns). This Table is linked to Fig 5.
(PDF)

S2 Table Effects of GE on the sensitivities of a panel of 9 virus strains to V2 mAbs and their ancestors. Neutralizing IC50s of V2 bnAbs and their ancestors were tested against 9 untreated and GE-modified 'V2 sensitive' viruses. The most sensitive modifications for each mAb and ancestor in each row are boxed. Geometric means are shown, omitting mAb-virus combinations where IC50s were >10μg/ml under all GE conditions. This Table is linked to Fig 8C.
(PDF)
