# Supplementary figures and images for: Glycoengineering HIV-1 Env creates ‘supercharged’ and ‘hybrid’ glycans to increase neutralizing antibody potency, breadth and saturation
Source: PLoS Pathog. 2018 May 2;14(5):e1007024. doi: 10.1371/journal.ppat.1007024 (PMC5951585; doi:10.1371/journal.ppat.1007024)

### A) JR-FL

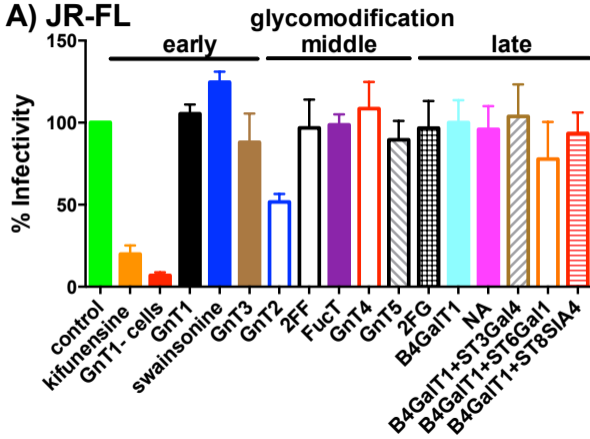

### B) BG505

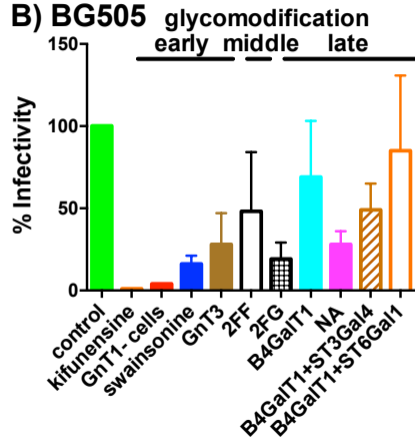

Supplement: S1 Fig — The effects of various GE treatments on A) JR-FL and B) BG505 PV infectivities were measured in CF2.CD4.CCR5 cells. Infectivity is shown relative to the untreated control, set to 100%, with treatments separated into those that affect early, middle and late stages of the N-linked glycosylation pathway. NA treatment involved a 37°C incubation for 1h followed by a PBS wash. The resulting PV and a mock (no enzyme) incubation control both had largely undiminished infectivities as compared to the "no treatment" control. All assays were repeated at least 6 times. Error bars show SD. (PDF) [file ppat.1007024.s001.pdf]

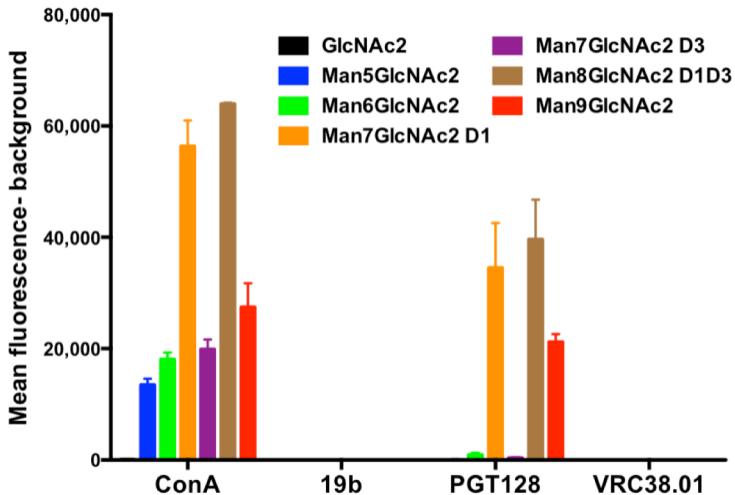

Supplement: S3 Fig — 33μM of GlcNAc2 and various oligomannose glycans were printed and checked for binding by 50μg/mL of various mAbs and Concanavalin A. Fluorescence was background-subtracted using the local method in GenePix Pro7 software. Means and standard errors of 6 replicates are shown from two independent tests. (PDF) [file ppat.1007024.s003.pdf]

# JR-FL gp160 $\Delta$ CT

% Residual infectivity

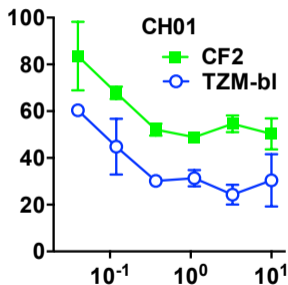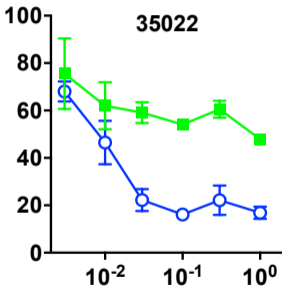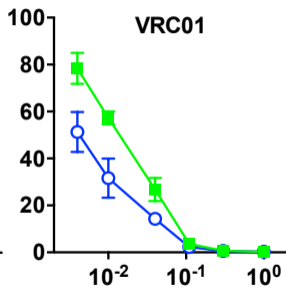

mAb concentration (μg/ml)

Supplement: S4 Fig — The neutralizing activities of mAbs CH01, 35O22 and VRC01 against the JR-FL E168K+N189A PV were compared in the TZM-bl and CF2 assays. All assays were repeated at least 3 times in duplicate. Error bars show SD. (PDF) [file ppat.1007024.s004.pdf]

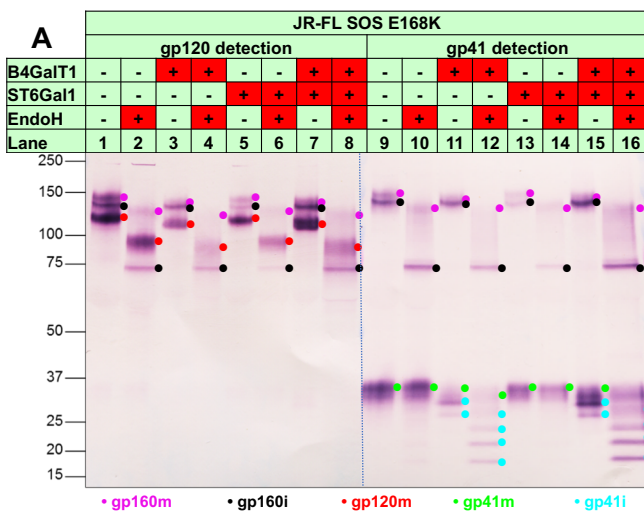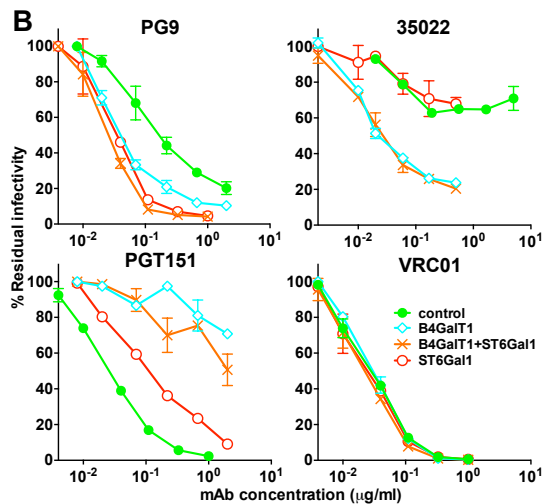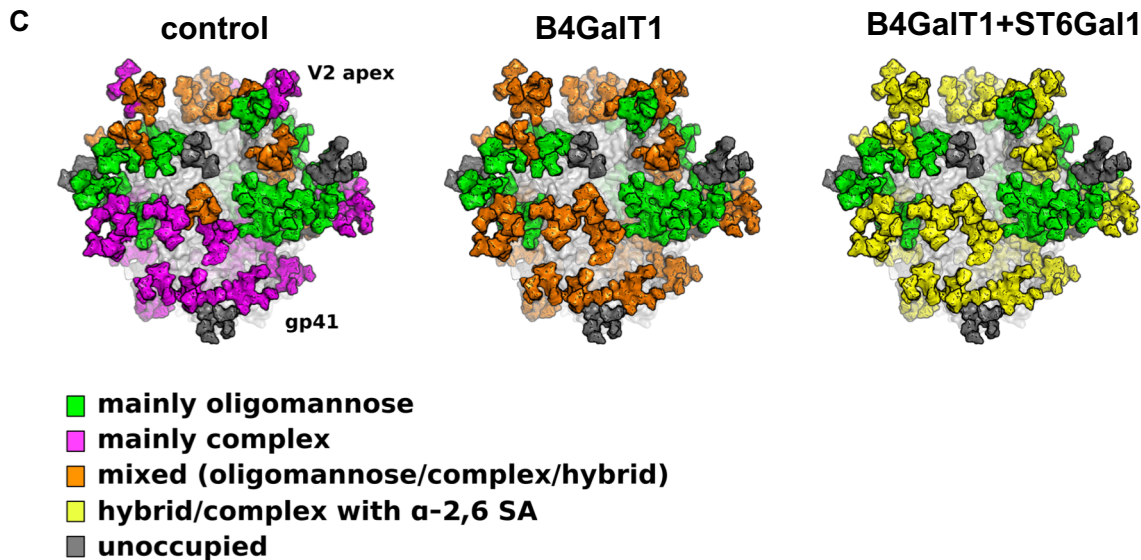

Supplement: S5 Fig — The effects of B4GalT1 and ST6Gal1 alone and together on JR-FL Env was assessed. A) Changes in the mobility of JR-FL SOS E168K gp160ΔCT Env produced with the indicated plasmid co-transfections were or were not treated with endo H and then analyzed by SDS-PAGE-Western blot, with or without endo H treatment. Dots indicate Env species, as in Fig A and B of S1 Text. B) Sensitivities of GE-modified JR-FL PVs to mAbs PG9, 35O22, PGT151 and VRC01. Results are representative of two repeats performed in duplicate. Error bars show SD. C) Model of the effects of GE on JR-FL Env trimers. The glycosylated JR-FL 5FUU structure [3] was modeled, with glycans colored coded, as in a previous JR-FL SOSIP trimer analysis [23], to illustrate how B4GalT1 overexpression drives the conversion of complex glycans (magenta) to form hybrid glycans (orange) and that B4GalT1+ST6Gal1 expression drives α-2,6 sialylation of these glycans (yellow). Glycan assignments are for illustrative purposes only. (PDF) [file ppat.1007024.s005.pdf]

Non-nAbs

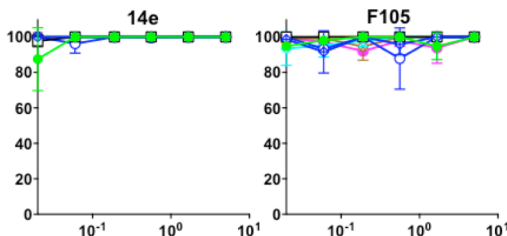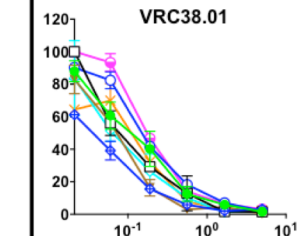

V2 apex

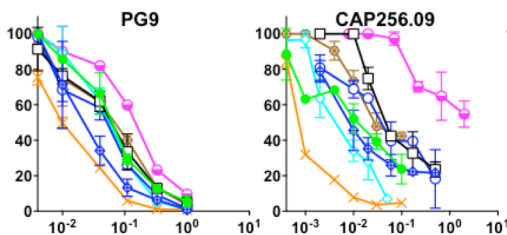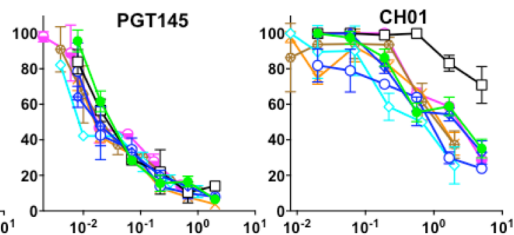

V3 glycan supersite

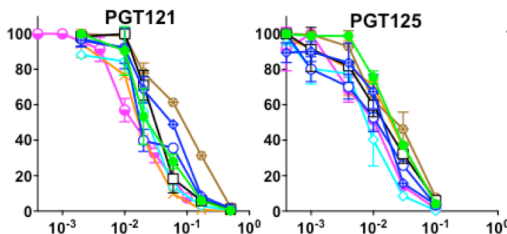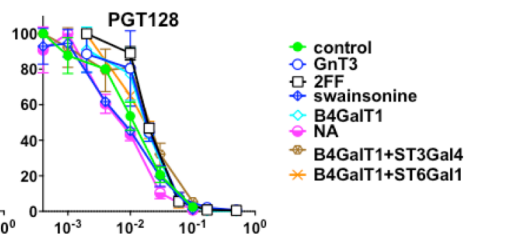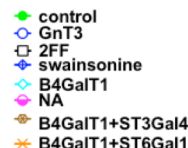

CD4bs

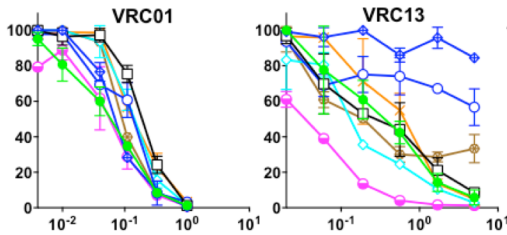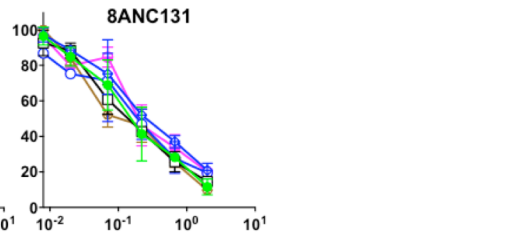

Gp120-gp41 interface

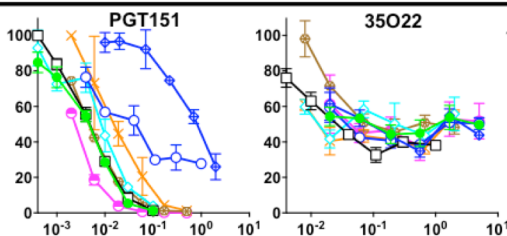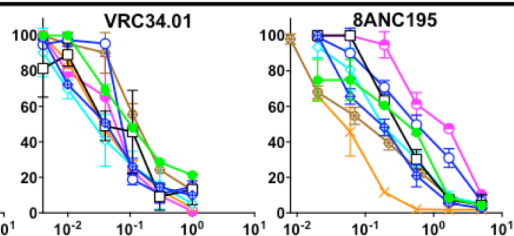

MPER

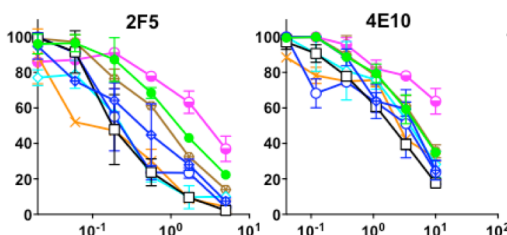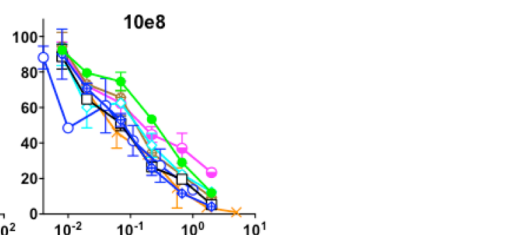

mAb concentration ( $\mu\text{g/ml}$ )

Supplement: S6 Fig — The effects of modifying selected steps in BG505 T332N WT trimer glycan maturation on mAb sensitivity was assessed in a manner analogous to Fig 3. Kifunensine treatment and GnT1- PV had infectivities too low to be measured reliably and were therefore omitted. Results are representative of two repeats performed in duplicate. Error bars show SD. IC50s are shown in Fig 2B. (PDF) [file ppat.1007024.s006.pdf]

● control

✕ B4GalT1+ST6Gal1

□ BG505 K169E no trt

△ BG505 K169E B4GalT1+ST6Gal1

**A****BG505****CAP256 plasma**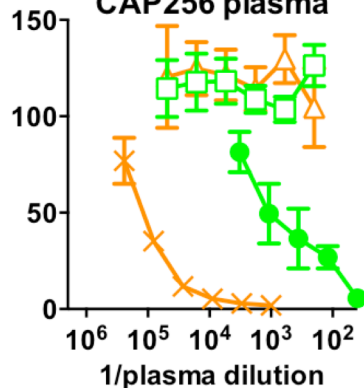**B****JR-FL****N152 plasma**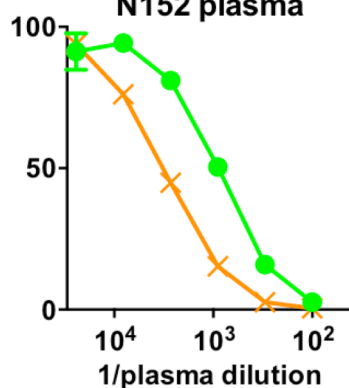**CAP256.09**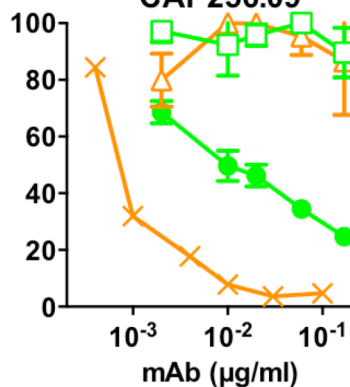**35022**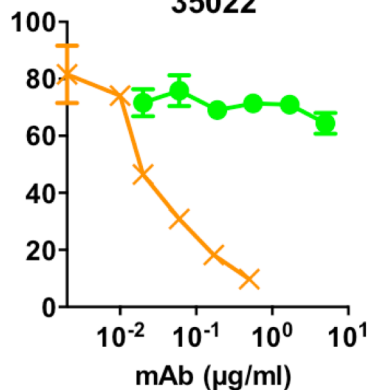

Supplement: S7 Fig — A) Sensitivities of control and B4GalT1+ST6Gal1-modified BG505 PV with or without a K169E knockout mutation (to knock out CAP256.09 lineage binding) to plasmas from infected donor CAP256 and CAP256.09, a bnAb isolated from CAP256 donor. B) Comparison of the sensitivities of control and B4GalT1+ST6Gal1 JR-FL PVs to plasma from infected donor N152 and 35O22, a bnAb isolated from N152 donor. Results are representative of two repeat assays performed in duplicate; error bars show SD. (PDF) [file ppat.1007024.s007.pdf]

% Residual infectivity

## A JR-FL

PG9

PGT151

35022

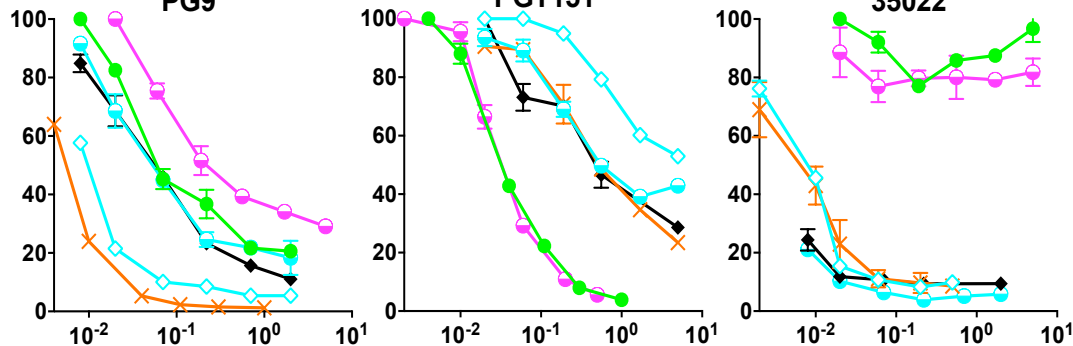

## B BG505

CAP256.09

PGT151

8ANC195

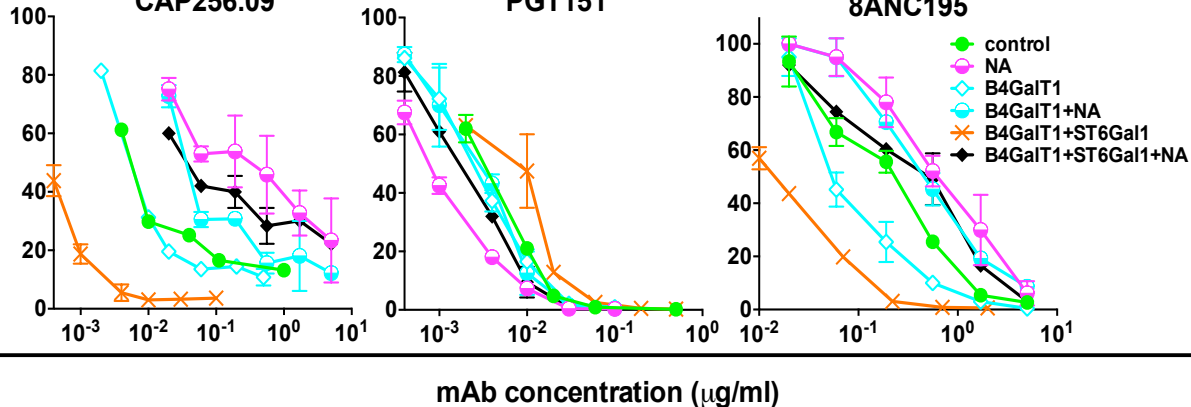

Supplement: S8 Fig — Changes in sensitivity of glycomodified JR-FL (A) and BG505 PVs (B) after NA digestion were assessed. PV produced with co-transfected B4GalT1 alone and B4GalT1+ST6Gal1 were compared with the untreated control, with or without NA digestion. Results are representative of at least two repeats performed in duplicate. Error bars represent standard deviations. (PDF) [file ppat.1007024.s008.pdf]

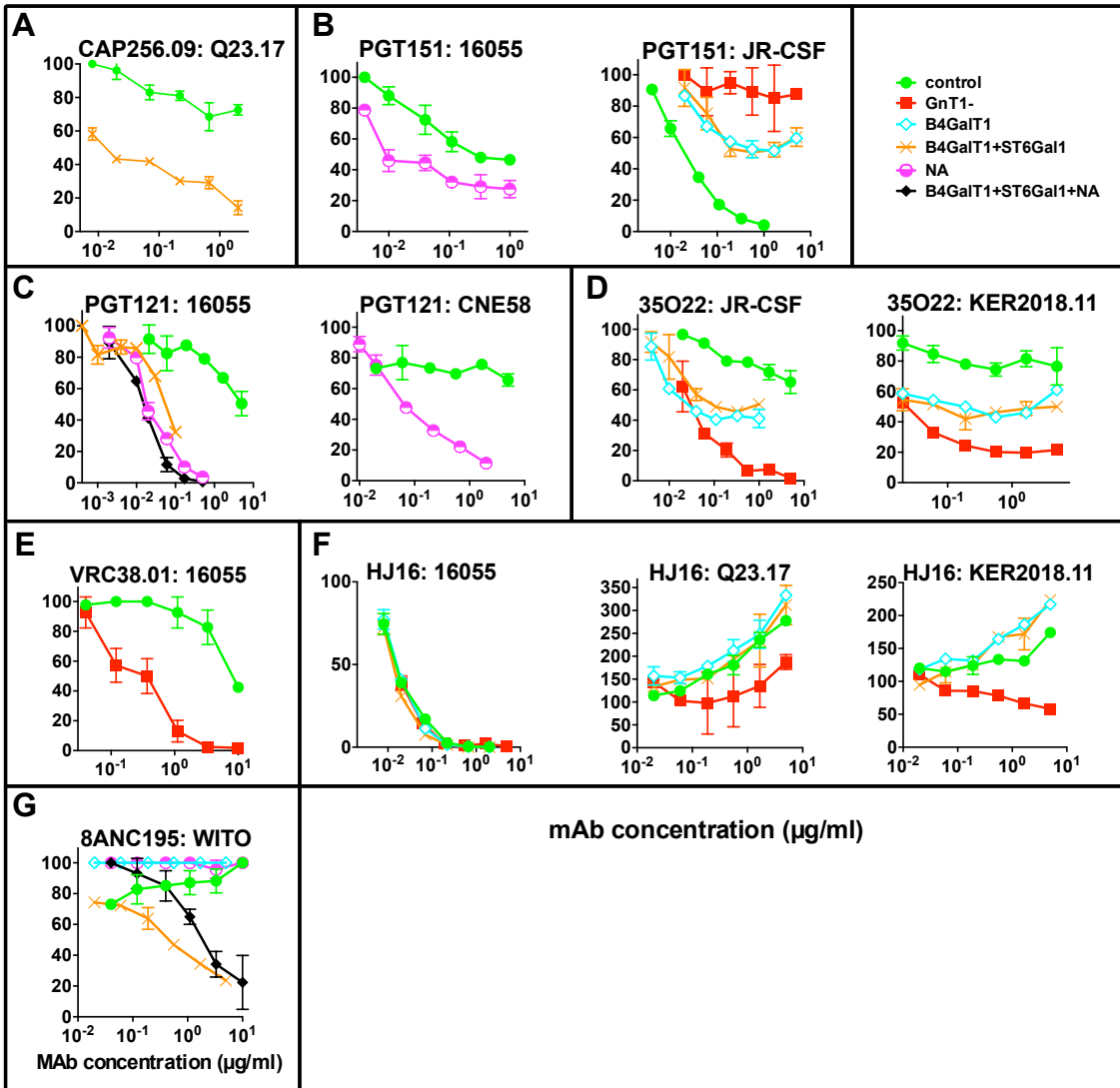

Supplement: S10 Fig — MAb titrations against different virus strains under various conditions show some of the titrated effects summarized as IC50s in Fig 5. All assays were repeated at least 2 times in duplicate. Error bars show SD. (PDF) [file ppat.1007024.s010.pdf]

% Residual infectivity

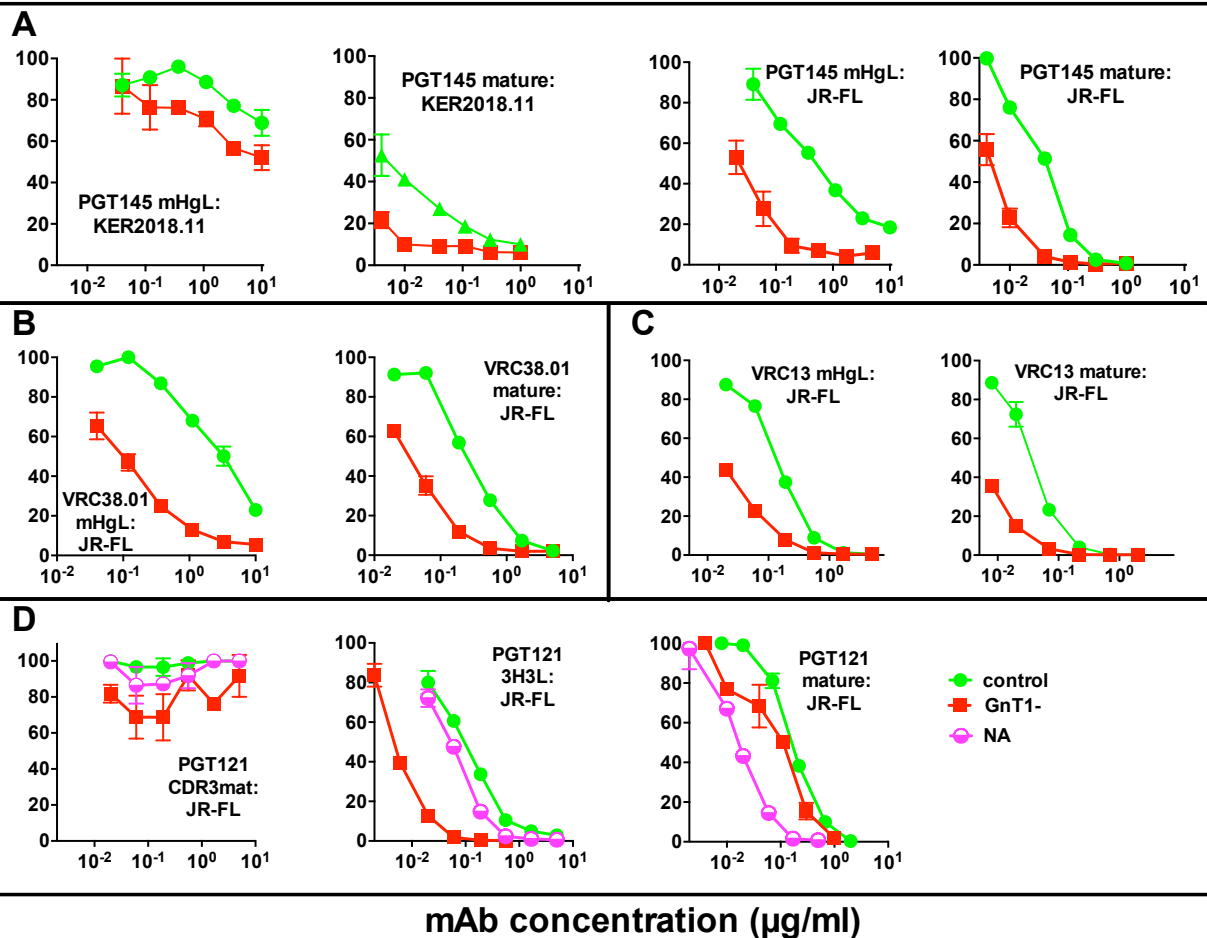

Supplement: S12 Fig — A) Sensitivities of control and GnT1- modified KER2018.11 and JR-FL WT PVs to mature PGT145 and its mHgL ancestor; B) Sensitivities of control and GnT1- modified JR-FL WT to mature VRC38.01 and its mHgL ancestor; C) Sensitivities of control and GnT1- modified JR-FL WT to mature VRC13 and its mHgL ancestor; D) Sensitivities of control, NA-treated and GnT1- JR-FL WT PVs to neutralization by mature PGT121 and its ancestors 3H3L and CDR3mat. (PDF) [file ppat.1007024.s012.pdf]
