# Supplementary material for: Glycoengineering HIV-1 Env creates ‘supercharged’ and ‘hybrid’ glycans to increase neutralizing antibody potency, breadth and saturation
Source: PLoS Pathog. 2018 May 2;14(5):e1007024. doi: 10.1371/journal.ppat.1007024 (PMC5951585; doi:10.1371/journal.ppat.1007024)
Supplement: S2 Fig — Results are representative of at least two repeats performed in duplicate performed in duplicate. Error bars show standard deviations (SD). IC50s are shown in Fig 2. (PDF) [file ppat.1007024.s002.pdf]

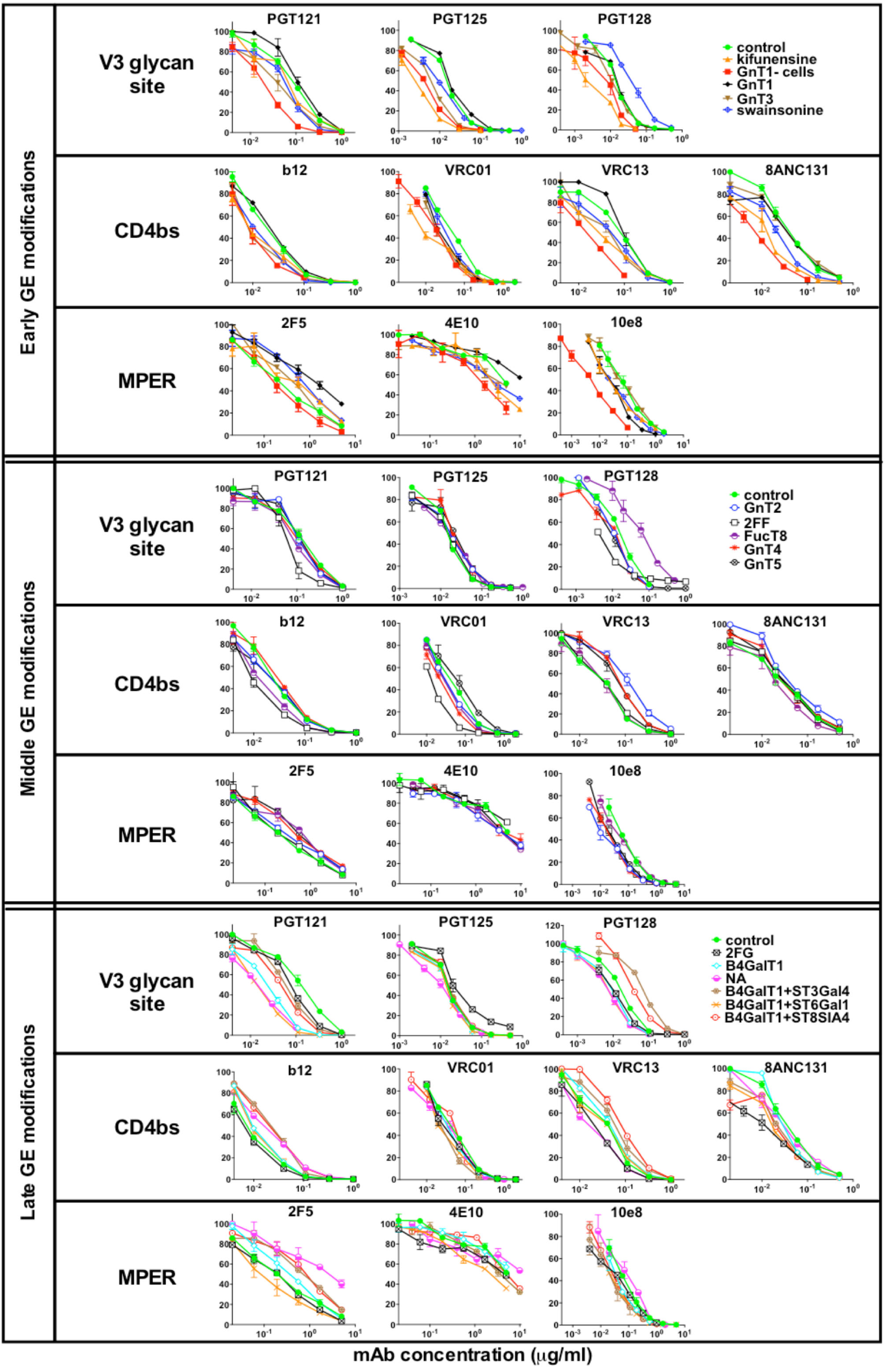
% Residual infectivity
Early GE modifications
Middle GE modifications
Late GE modifications
V3 glycan site
CD4bs
MPER
PGT121
PGT125
PGT128
b12
VRC01
VRC13
8ANC131
2F5
4E10
10e8
control
kifunensine
GnT1- cells
GnT1
GnT3
swainsonine
GnT2
2FF
FucT8
GnT4
GnT5
2FG
B4GalT1
NA
B4GalT1+ST3Gal4
B4GalT1+ST6Gal1
B4GalT1+ST8SIA4
mAb concentration (μg/ml)
