# Supplementary material for: Glycoengineering HIV-1 Env creates ‘supercharged’ and ‘hybrid’ glycans to increase neutralizing antibody potency, breadth and saturation
Source: PLoS Pathog. 2018 May 2;14(5):e1007024. doi: 10.1371/journal.ppat.1007024 (PMC5951585; doi:10.1371/journal.ppat.1007024)
Supplement: S9 Fig — The effects of B4GalT1 co-transfection on Envs from 14 different strains were compared by SDS-PAGE-Western blot. VLPs expressed A) without or B) with co-transfected B4GalT1 were loaded at equal concentrations without or with endo H treatment and probed with an anti-gp41 primary mAb cocktail (2F5 and 4E10). Dots denote different Env species, as in Fig A and B of S1 Text. (PDF) [file ppat.1007024.s009.pdf]

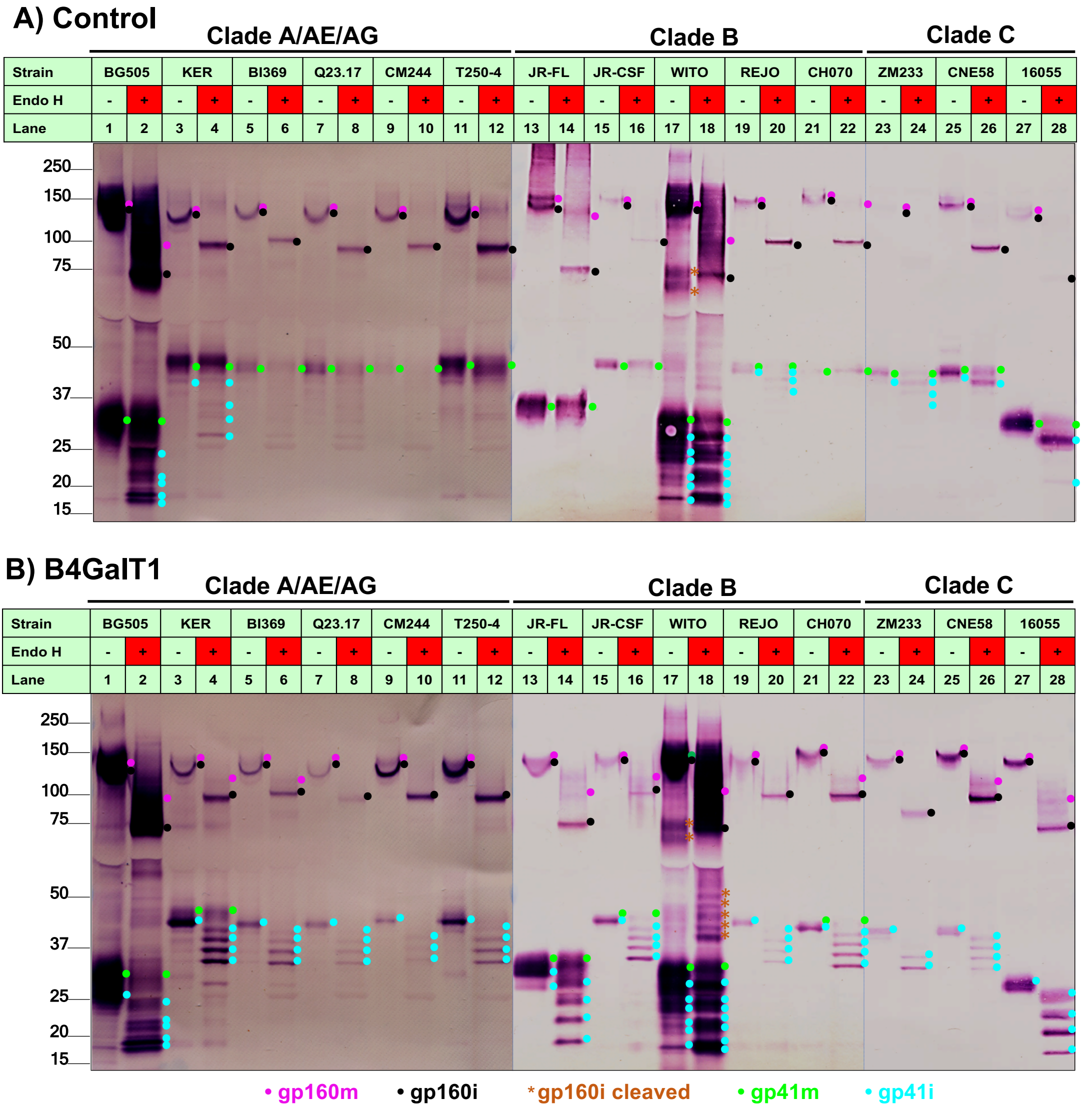
A) Control
Clade A/AE/AG
Clade B
Clade C
Strain
BG505
KER
BI369
Q23.17
CM244
T250-4
JR-FL
JR-CSF
WITO
REJO
CH070
ZM233
CNE58
16055
Endo H
- + - + - + - + - + - + - + - + - + - + - + - + - + - +
Lane
1 2 3 4 5 6 7 8 9 10 11 12 13 14 15 16 17 18 19 20 21 22 23 24 25 26 27 28
250
150
100
75
50
37
25
20
15
B) B4GalT1
Clade A/AE/AG
Clade B
Clade C
Strain
BG505
KER
BI369
Q23.17
CM244
T250-4
JR-FL
JR-CSF
WITO
REJO
CH070
ZM233
CNE58
16055
Endo H
- + - + - + - + - + - + - + - + - + - + - + - + - + - +
Lane
1 2 3 4 5 6 7 8 9 10 11 12 13 14 15 16 17 18 19 20 21 22 23 24 25 26 27 28
250
150
100
75
50
37
25
20
15
• gp160m
• gp160i
* gp160i cleaved
• gp41m
• gp41i
