# Supplementary material for: Glycoengineering HIV-1 Env creates ‘supercharged’ and ‘hybrid’ glycans to increase neutralizing antibody potency, breadth and saturation
Source: PLoS Pathog. 2018 May 2;14(5):e1007024. doi: 10.1371/journal.ppat.1007024 (PMC5951585; doi:10.1371/journal.ppat.1007024)
Supplement: S1 Text — (PDF) [file ppat.1007024.s013.pdf]

S1 Text: SDS-PAGE-Western blot reveals B4GalT1-induced glycan "thinning"

To further contextualize the JR-FL neutralization data, GE-modified VLP gp120 and gp41 were analyzed in separate SDS-PAGE-Western blots. In Figs A and B, Env species are marked by colored dots and were identified by comparing the patterns in separate blots probed with gp120 or gp41 mAbs. Endo H treatments were used to parse endo H-sensitive high mannose/hybrid glycans from endo H-resistant complex glycans (see Fig 1). Endo H-resistant species were termed "mature" (m). Endo H-sensitive species were termed "immature" (i). The various species in Figs A and B were previously identified in a similar SDS-PAGE analysis (see Fig 4 in [1]).

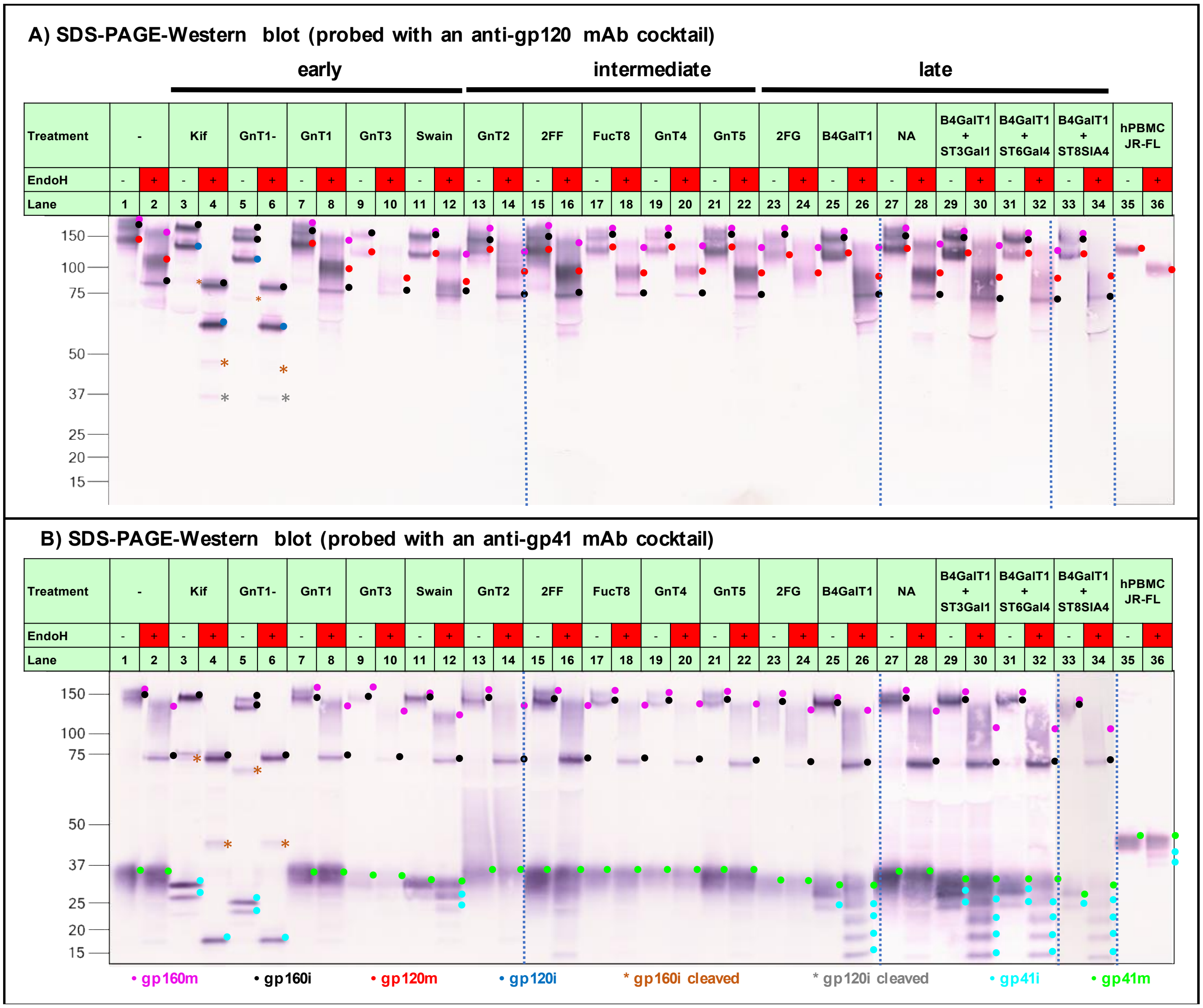

Although most GE treatments had minor effects, there were some notable exceptions. As expected, kifunensine and GNT1- Env bands were sharper and ran faster than controls, reflecting homogeneous, endo H-sensitive high mannose glycans (Fig A and B, lanes 1-6). As expected, GNT1- gp120 and gp41 bands migrated faster than their kifunensine counterparts (Fig A and B, lanes 3 and 5), but coalesced upon endo H treatment. The two GNT1- gp160 bands (Fig A and B, lane 5) may be trimmed (e.g. $Man_5GlcNAc_2$) and untrimmed (e.g. $Man_9GlcNAc_2$) glycoforms. The two gp41 bands of GnT1- and kifunensine virus suggest possible incomplete occupation of gp41 sequons (Fig B, lanes 3 and 5). Several other treatments (GNT3, swainsonine and B4GalT1+/-various sialyltransferases) increased Env's endo H sensitivity (Fig A and B, compare lanes 1, 2, 9-12, 15, 16, 25, 26 and 29-34). Of these, treatments that included B4GALT1 had the most profound effects, where, upon endo H digestion, a gp41 laddering effect was observed. This suggests that complex glycans were partially replaced with hybrid glycans, andf appears to account for the unexpected effects of B4GALT1 on PV sensitivities to interface bnAbs PGT151 and 35O22 activities (Fig 2). Coexpression of ST3GAL4, ST6GAL1 or ST8SIA4 with

B4GalT1 did not cause further changes. GNT3, swainsonine and 2FF also partially converted complex glycans to hybrid glycans, but less effectively than B4GALT1.

Other GE treatments had relatively modest effects on Env mobility, consistent with our findings above. However, 2FG-modified gp120 and gp41 migrated relatively fast (Fig A and B, lanes 23 and 24), consistent with reduced galactosylation. However, NA gp120 and gp41 migrated similarly to controls (Fig A and B, lanes 27 and 28) - removing negatively charged SA has no impact here, since the negative charge for protein migration is largely imparted by SDS.

PBMC-derived gp120 migrated similarly to the VLP gp120 control and was endo H-resistant (Fig A, compare lanes 1 and 2 with lanes 35 and 36). Uncleaved gp160 and high mannose gp120 were not detected. PBMC-derived gp41 was largely endo H-resistant and migrated far slower than controls, consistent with the presence of an additional ~16.3 kDa gp41 cytoplasmic tail (Fig B, lanes 35 and 36).

1. Crooks ET, Tong T, Osawa K, Binley JM. Enzyme digests eliminate nonfunctional Env from HIV-1 particle surfaces, leaving native Env trimers intact and viral infectivity unaffected. J Virol. 2011;85(12):5825-39. doi: 10.1128/JVI.00154-11. PubMed PMID: 21471242; PubMed Central PMCID: PMC3126298.
