# Supplementary material for: Glycoengineering HIV-1 Env creates ‘supercharged’ and ‘hybrid’ glycans to increase neutralizing antibody potency, breadth and saturation
Source: PLoS Pathog. 2018 May 2;14(5):e1007024. doi: 10.1371/journal.ppat.1007024 (PMC5951585; doi:10.1371/journal.ppat.1007024)
Supplement: S2 Text — (PDF) [file ppat.1007024.s014.pdf]

S2 Text. Further investigation of interface bnAbs, V2 bnAb lineage members and poly SA termini.

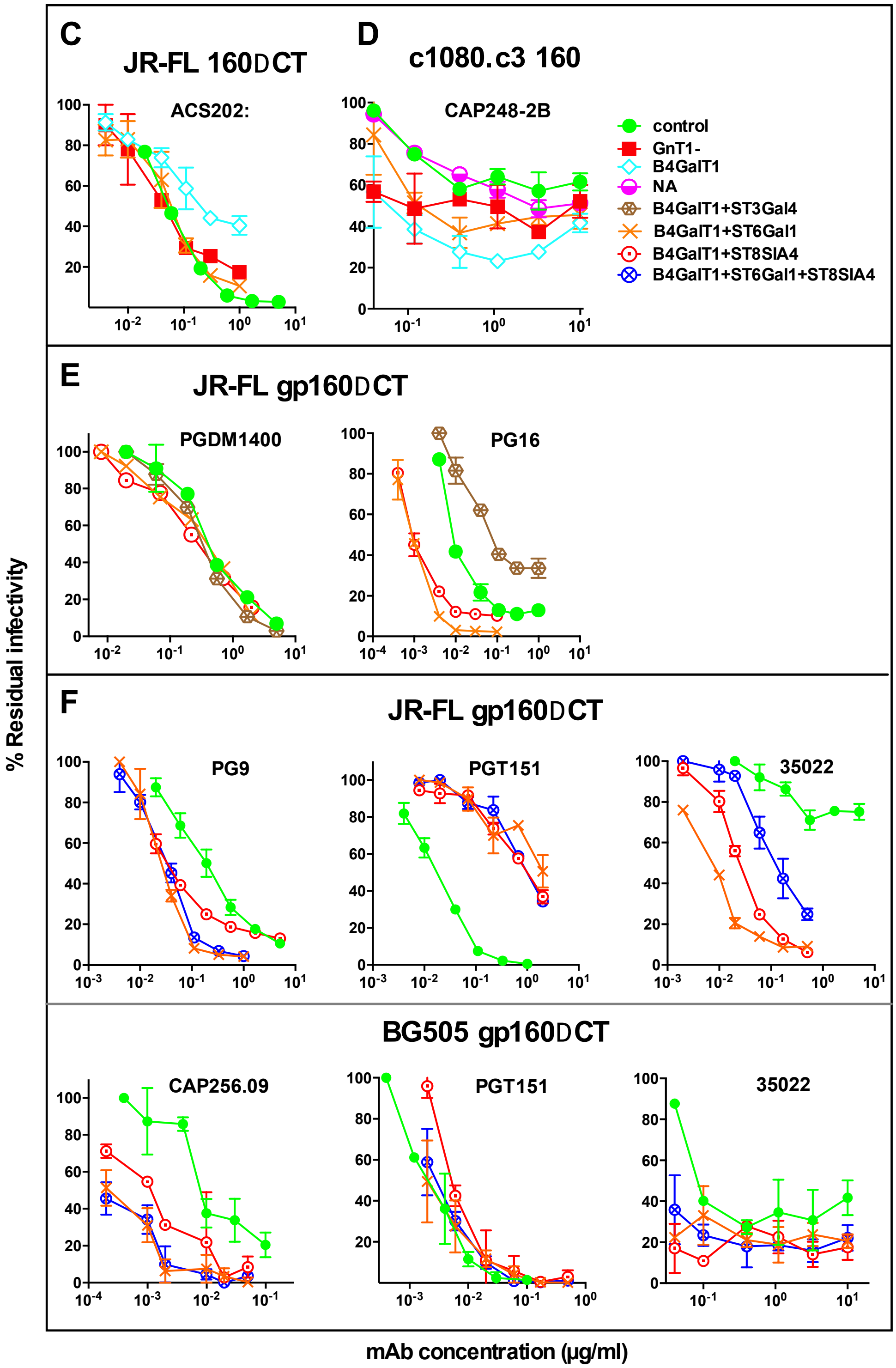

We examined the effects of GE on recently reported interface bnAbs ACS202 and CAP248-2B [65, 69]. ACS202 was largely unaffected, except for a slight loss of activity against the B4GALT1 virus (Fig C). CAP248-2B neutralization saturated at <50% but was improved against GnT1- and B4GALT1+/-ST6Gal1-modified viruses (Fig D), suggesting that smaller glycans may resolve clashes.

To determine whether the behaviors of PG9 and PGT145 above were consistent within their developmental lineages, we tested their relatives PG16 and PGDM1400, respectively on GE-modified JR-FL (Fig E). Like PG9 (Fig 3), PG16 was more effective against B4GALT1+ST6GAL1 and B4GALT1+ST8SIA4 viruses but was weaker against the B4GALT1+ST3Gal4 virus. Like PGT145, PGDM1400 was unaffected by GE.

Poly-SA termini do not improve V2 apex or interface bnAb sensitivities.

We next investigated the possibility that further increasing charge of ST6GAL1-modified virus via poly-SA chains might further impact sensitivity to V2 apex and interface bnAbs. This possibility may have been overlooked by using B4GALT1+ST8SIA4 above (Fig 3), as this GE treatment lacks ST6Gal1 that might be important to provide α-2,6 SA termini as substrates for ST8SIA4. However, this triple treatment had little effect on JR-FL sensitivities to PG9 or PGT151, as compared to controls (Fig F, upper panels). Conversely, the triple treatment blunted the 35O22 enhanced activity observed with the double treatments, suggesting that the additional bulk of α-2,8-linked SAs clashes with this bnAb. The effects on BG505 sensitivity to CAP256.09 were similar to those on JR-FL sensitivity to PG9 (Fig F). As expected, the triple treatment had little impact on either PGT151 or 35O22 neutralization of BG505, as for the other GE treatments (Fig F).
