# Supplementary material for: Glycoengineering HIV-1 Env creates ‘supercharged’ and ‘hybrid’ glycans to increase neutralizing antibody potency, breadth and saturation
Source: PLoS Pathog. 2018 May 2;14(5):e1007024. doi: 10.1371/journal.ppat.1007024 (PMC5951585; doi:10.1371/journal.ppat.1007024)
Supplement: S1 Table — MAb neutralization sensitivities of a 14-virus panel was tested using select GE modifications. The most sensitive GE modification for each mAb-strain combination is boxed. Some GE PV infectivities were too low to be measured reliably, denoted as ND (not determined). Geometric means are shown, omitting IC50s >10μg/ml under all GE conditions for a particular mAb-virus combination. Wilcoxon Signed Rank tests were performed on data for each mAb-PV pair organized into two columns to compare IC50s under control and GE conditions. p values showing significant increased or decreased sensitivity are shown by blue or red asterisks, respectively, or were not significant (ns). This Table is linked to Fig 5. (PDF) [file ppat.1007024.s015.pdf]

Modification	Clade/Strain	A				AE	AG	B				BC	C			
		BG505	KER2018	BI369.9A	Q23.17	CM244.ec1	T250-4	JR-FL	JR-CSF	WITO	REJO	CH070.1	ZM233.6	CNE58	16055	Geo Mean
PG9	Control	0.080	0.009	0.070	0.010	0.001	0.003	0.300	0.006	0.010	0.030	0.009	0.010	0.060	0.050	0.017
	B4GalT1	0.050	0.009	0.005	0.004	0.010	0.002	0.030	0.003	0.003	0.002	0.010	0.003	0.030	0.010	0.007
	ST6Gal1	0.006	0.005	0.005	0.002	0.010	0.002	0.030	0.001	0.008	0.002	0.007	0.003	0.010	0.006	0.005
	B4GalT1+ST6Gal1	0.020	0.005	0.010	0.003	0.004	0.001	0.020	0.0009	0.0004	0.003	0.002	0.001	0.005	0.008	0.003
	NA	0.300	0.050	0.100	0.020	0.010	0.008	1.0	0.020	0.020	0.040	0.030	0.030	0.070	0.200	0.049
CAP256.09	Control	0.007	0.004	0.005	3.0	0.130	0.002	10	10	10	10	0.020	0.0008	0.005	0.004	0.011
	B4GalT1	0.002	0.002	0.001	3.0	0.100	0.001	10	10	10	10	0.006	0.0002	0.0008	0.001	0.004
	ST6Gal1	0.0003	0.0002	0.0002	10	0.020	0.0002	10	10	10	10	0.0009	0.0002	0.0006	0.001	0.001
	B4GalT1+ST6Gal1	0.0002	0.0003	0.0004	0.010	0.020	0.0002	10	10	10	10	0.0003	0.00008	0.0006	0.0003	0.001
	NA	3.0	0.009	0.050	10	10	0.004	10	10	10	10	10	0.040	0.010	0.010	0.171
PGT151	Control	0.009	0.080	0.020	0.030	10	0.008	0.020	0.020	0.030	0.030	10	0.010	0.010	0.200	0.023
	GnT1-	ND	10	ND	ND	10	0.070	0.40	10	10	0.120	10	10	ND	10	2.1
	B4GalT1	0.020	1.0	0.32	2.0	10	0.070	2.0	10	10	0.51	10	10	0.080	10	0.97
	B4GalT1+NA	0.008	0.090	0.220	0.050	10	0.020	0.50	0.006	10	0.280	10	10	0.120	10	0.224
	B4GalT1+ST6Gal1	0.040	1	5.0	0.40	10	0.030	0.80	10	10	0.210	10	10	0.100	10	0.93
	NA	0.003	0.020	0.010	0.010	10	0.005	0.01	0.010	0.020	0.070	10	0.006	0.006	0.004	0.010
PGT121	Control	0.050	10	0.020	0.006	10	0.020	0.100	0.040	0.67	10	0.020	0.060	10	3.0	0.205
	GnT1-	ND	10	0.010	0.007	10	0.020	0.020	0.060	2.0	0.050	0.130	10	ND	10	0.201
	B4GalT1	0.050	10	0.008	0.005	10	0.006	0.040	0.030	0.260	0.070	0.020	0.150	0.100	0.090	0.057
	B4GalT1+ST6Gal1	0.020	10	0.007	0.002	10	0.003	0.010	0.020	0.130	0.050	0.009	0.53	0.160	0.080	0.040
	B4GalT1+NA	0.009	0.42	0.001	0.002	10	0.002	0.010	0.009	0.090	0.007	0.006	0.040	0.100	0.010	0.013
	NA	0.008	10	0.003	0.001	10	0.004	0.010	0.010	0.090	0.130	0.010	0.040	0.040	0.004	0.020
35022	Control	10	10	10	10	10	10	10	10	10	10	10	10	10	10	10
	GnT1-	ND	0.010	0.009	0.010	0.006	0.010	0.008	0.030	0.090	0.004	0.080	0.030	ND	0.150	0.019
	B4GalT1	10	10	0.005	0.020	0.005	10	0.020	0.030	10	0.002	10	0.004	2.0	10	0.192
	B4GalT1+ST6Gal1	10	10	0.010	10	0.002	10	0.010	0.200	5.0	0.009	10	0.020	2.0	10	0.40
CH01	Control	7	0.300	0.940	0.050	0.080	0.060	10	10	10	10	10	10	0.160	10	1.2
	GnT1-	ND	0.030	0.040	0.003	0.020	0.020	0.040	0.090	0.030	10	0.600	0.005	ND	10	0.029
PGT145	Control	0.060	0.004	5.0	0.80	0.002	0.001	0.060	0.002	0.004	0.005	0.020	2.0	0.120	0.015	0.029
	GnT1-	ND	0.001	0.007	10	0.0008	0.001	0.008	0.002	0.008	0.001	0.003	0.0005	ND	0.002	0.004
	B4GalT1	0.030	0.020	0.009	3.0	0.0007	0.0009	0.050	0.003	0.002	0.001	0.050	0.006	0.030	0.140	0.013
	B4GalT1+ST6Gal1	0.020	0.030	0.040	0.50	0.001	0.002	0.060	0.003	0.001	0.001	0.010	0.004	0.020	0.100	0.011
	B4GalT1+NA	0.003	0.009	0.006	10	0.002	0.0008	0.020	0.0007	0.0008	0.0004	0.010	0.003	0.005	0.002	0.005
	NA	0.010	0.020	0.010	3.0	0.001	0.002	0.020	0.001	0.003	0.001	0.030	10	0.010	0.010	0.015
VRC13	Control	0.40	0.030	0.020	0.080	0.030	10	0.050	0.050	0.110	0.030	10	0.020	0.020	0.150	0.076
	GnT1-	ND	0.070	0.004	0.010	0.008	10	0.009	0.009	0.060	0.010	10	0.100	ND	0.300	0.039
	B4GalT1	0.100	2.0	0.006	0.050	0.030	10	0.040	0.030	10	0.040	10	0.010	0.020	0.110	0.105
	B4GalT1+ST6Gal1	0.80	1.0	0.070	0.100	0.080	0.200	0.030	0.060	10	0.020	10	0.030	0.030	0.100	0.130
	B4GalT1+NA	0.090	0.050	0.010	0.030	0.090	0.030	0.040	0.009	10	0.005	10	0.010	0.020	0.040	0.039
	NA	0.030	0.070	0.010	0.030	0.010	0.43	0.020	0.020	0.100	0.006	10	0.009	0.010	0.040	0.026
VRC38	Control	0.200	0.060	1.1	0.01	1.0	0.070	0.280	0.320	0.020	3.0	10	10	0.59	6.0	0.273
	GnT1-	ND	0.015	ND	0.005	0.280	0.160	0.030	0.180	0.020	0.050	10	10	ND	0.240	0.055
	B4GalT1+ST6Gal1	0.200	0.020	0.85	0.004	1.1	0.140	0.200	0.150	0.010	0.200	10	10	0.230	1.9	0.140
8ANC195	Control	0.40	10	10	10	10	10	10	10	10	ND	10	10	10	10	2.0
	B4GalT1	0.140	10	10	10	10	10	10	10	10	ND	10	10	10	10	1.2
	B4GalT1+ST6Gal1	0.022	10	10	10	10	10	10	10	0.220	ND	10	10	10	10	0.070

≤0.003
0.004 - 0.010
0.011 - 0.030
0.031 - 0.100
0.110 - 0.300
>0.300

p values for Wilcoxon Signed Rank tests on mAb IC50s against GE viruses compared to control untreated viruses

	PG9	CAP256.09	PGT151	PGT121	35022	CH01	PGT145	VRC13	VRC38.01
ST6Gal1	**	ns							
B4GalT1	**	**	***	*	**		ns	ns	
B4GalT1+ST6Gal1	***	**	***	*	**		ns	ns	*
NA	***	**	*	***			ns	**	
B4GalT1+NA			**	***			ns	ns	
GnT1-			**	ns	***	**	ns	ns	*

ns=not significant

* <=0.05

**<=0.01

***<=0.001

****<=0.0001

* = increased potency

* = reduced potency
