# Supplementary material for: Glycoengineering HIV-1 Env creates ‘supercharged’ and ‘hybrid’ glycans to increase neutralizing antibody potency, breadth and saturation
Source: PLoS Pathog. 2018 May 2;14(5):e1007024. doi: 10.1371/journal.ppat.1007024 (PMC5951585; doi:10.1371/journal.ppat.1007024)
Supplement: S2 Table — Neutralizing IC50s of V2 bnAbs and their ancestors were tested against 9 untreated and GE-modified 'V2 sensitive' viruses. The most sensitive modifications for each mAb and ancestor in each row are boxed. Geometric means are shown, omitting mAb-virus combinations where IC50s were >10μg/ml under all GE conditions. This Table is linked to Fig 8C. (PDF) [file ppat.1007024.s016.pdf]

Antibody	Version	Strain / Modification	ZM233.6	T250-4	16055	C1080.c3	Q23.17	WITO.33	KER2018	CAP256 week 34	CM244.ec1	Geo Mean
CAP256.09	Mature	Untreated	0.0008	0.002	0.004	10	3.0	10	0.004	0.003	0.130	0.027
		B4GalT1+ST6Gal1	0.00008	0.0002	0.0003	0.003	0.010	10	0.0003	0.0001	0.020	0.001
		GnT1-	0.006	0.020	0.010	10	10	10	0.015	0.010	0.80	0.105
	Ancestor I1	Untreated	10	9.5	10	10	10	10	10	0.080	10	1.966
		B4GalT1+ST6Gal1	10	9.0	9.5	10	10	10	10	0.030	10	1.369
		GnT1-	10	9.0	0.080	10	10	10	10	0.010	10	0.193
	UCA	Untreated	10	10	10	10	10	10	10	1.5	10	1.5
		B4GalT1+ST6Gal1	10	10	10	10	10	10	10	0.30	10	0.30
		GnT1-	10	10	10	10	10	10	10	0.40	10	0.40
PG9	Mature	Untreated	0.010	0.003	0.050	0.003	0.010	0.010	0.009	0.020	0.001	0.008
		B4GalT1+ST6Gal1	0.001	0.001	0.008	0.002	0.003	0.0004	0.005	0.006	0.004	0.002
		GnT1-	0.004	0.020	0.050	0.020	0.010	0.010	0.030	0.060	0.030	0.020
	Ancestor gHmL	Untreated	0.42	0.060	1.0	0.040	0.160	0.90	0.40	0.90	0.110	0.262
		B4GalT1+ST6Gal1	0.060	0.030	1.5	0.040	0.100	0.260	0.190	1.3	0.180	0.171
		GnT1-	0.020	0.040	0.50	0.120	0.020	1.0	0.160	0.43	0.060	0.117
CH04	Mature	Untreated	10	0.070	10	0.070	0.020	0.50	0.30	0.41	0.070	0.33
		GnT1-	0.008	0.020	0.5	0.060	0.005	0.050	0.01	0.070	0.020	0.029
	Ancestor RUA HC RUA LC	Untreated	10	10	10	2.0	0.010	4.0	10	10	10	0.95
		GnT1-	10	10	10	10	0.002	1.0	0.200	10	10	0.251
PGT145	Mature	Untreated	2.0	0.001	0.015	0.004	0.8	0.004	0.004	0.5	0.002	0.023
		GnT1-	0.0005	0.001	0.002	0.010	10	0.008	0.001	0.003	0.0008	0.005
	Ancestor mHgL	Untreated	10	0.5	10	10	10	0.010	10	10	0.060	0.234
		GnT1-	10	10	3.0	10	10	0.020	10	10	0.030	0.37
VRC38	Mature	Untreated	10	0.070	6.0	0.010	0.010	0.020	0.060	10	1	0.091
		GnT1-	10	0.160	0.240	0.010	0.005	0.020	0.015	10	0.280	0.040
	Ancestor mHgL	Untreated	10	0.150	10	0.47	0.100	0.130	0.230	10	10	0.184
		GnT1-	10	0.230	10	0.040	0.015	0.210	0.040	10	10	0.065

≤0.003
0.004 - 0.010
0.011 - 0.030
0.031 - 0.100
0.110 - 0.300
>0.300
